# A High Resolution Melting Analysis (HRM) PCR assay for the detection and identification of Old World *Leishmania* species

**DOI:** 10.1371/journal.pntd.0012762

**Published:** 2024-12-23

**Authors:** Yusr Saadi-Ben Aoun, Hejer Souguir, Hamed Chouaieb, Mongia Kraiem, Insaf Bel Hadj Ali, Ahmed S. Chakroun, Florian Noguier, Akila Fathallah-Mili, David Piquemal, Ikram Guizani

**Affiliations:** 1 Laboratory of Molecular Epidemiology and Experimental Pathology, Institut Pasteur de Tunis, Université de Tunis El Manar, Tunis, Tunisia; 2 Clinical Investigation Center, Institut Pasteur de Tunis, Tunis, Tunisia; 3 Institut National des Sciences Appliquées et de Technologie, Université de Carthage, Tunis, Tunisia; 4 Parasitology Department, Faculty of Medicine, Farhat Hached University Hospital, Université de Sousse, Sousse, Tunisia; 5 Acobiom, Montpellier, France; Tehran University of Medical Sciences, IRAN, ISLAMIC REPUBLIC OF

## Abstract

**Background:**

Cutaneous Leishmaniases (CL), highly endemic in Africa and Mediterranean region, are caused by different *Leishmania* parasite species. Accurate species identification is crucial for effective diagnosis, treatment, and control of these diseases, but traditionally relies on DNA-based methods. High Resolution Melting analysis PCR (HRM PCR) provides rapid results and precise differentiation based on nucleotide variations. We hypothesized that the *Strumpellin* gene of *Leishmania* could serve as an effective target for developing a HRM PCR method for the rapid and efficient detection and identification of *Leishmania* species in CL diagnosis.

**Methodology:**

The *Strumpellin* gene was investigated in Trypanosomatidae family using bioinformatics and phylogenetic approaches to explore its evolutionary conservation and suitability for HRM PCR. HRM PCR target and primers were selected and validated on 73 different *Leishmania* DNAs. The analytical limit of detection was assessed, and the performance for detecting and identifying parasites in 38 cutaneous lesions aspirates was compared to Direct Examination (DE) and ITS1-PCR RFLP methods.

**Findings:**

The developed HRM PCR assay accurately identified promastigote DNAs of *L. donovani/L. infantum, L. major*, *L. aethiopica*, *L. turanica, L. arabica*, *L. tarentolae* and 3 genotypes of *L. tropica.* Differentiation was achievable with as little as a single nucleotide difference occurring within or between species. HRM profile interpretations were consistent with sequencing results of the HRM PCR target and identification by ITS1-PCR RFLP. The assay could detect the equivalent of 24 *Leishmania* parasites. In a small-scale sample, we brought proof of principle demonstration the HRM could detect and identify *Leishmania* in human cutaneous samples. In comparison to DE, the sensitivity and specificity of the HRM PCR assay on human cutaneous samples were 88% and 84.62%, respectively, while the ITS1-PCR assay evaluation parameters were 84% and 92.31%. Statistical analysis confirmed good correlation among the three tests, with both molecular methods providing congruent parasite identification. Notably, in three samples, only the HRM PCR assay was able to assign them to *L. infantum* or *L. tropica.*

**Conclusions:**

The HRM PCR assay is a valuable tool for the detection and identification of Old World *Leishmania* species. Its integration into molecular diagnostic algorithms for CL or in eco-epidemiological studies holds promise for improving disease management and control.

## Author summary

Cutaneous Leishmaniases (CL) are a group of diseases considered as the most Neglected Tropical Diseases. CL present a complex epidemiology situation in many countries over the World and particularly in Africa and Middle East regions with the proven implication of different *Leishmania* species causing pleomorphic clinical presentations. Migrations, climate change and variations, agricultural developments are factors complicating eco-epidemiology and driving emerging trends. *Leishmania* species identification is pivotal for etiological diagnosis and patients’ management, and to establish adequate control measures. Molecular techniques are the most accurate methods for detection and identification of *Leishmania* parasites due to their higher sensitivity and specificity. HRM PCR assay constitutes a valuable test for rapid and reliable diagnosis of infectious diseases including CL. The present study aimed at the development of an HRM PCR targeting the *Strumpellin* gene, which would be useful for accurate CL diagnosis by detecting and identifying causal *Leishmania* parasites. Bioinformatic analyses explored conserved regions flanking interspecific polymorphisms to select HRM PCR target fragment and primer pairs and develop the assay. We confirmed through this study the relevance of this target and of our new method for CL diagnosis by proving the principle of using it to simultaneously detect and identify *Leishmania* parasites in human samples, thereby its potential to improve CL-patient management, surveillance and control in well-equipped health centers.

## Introduction

Cutaneous Leishmaniases (CL) are the most common forms of Leishmaniases. They are highly endemic in many countries, particularly in Africa and Mediterranean region [1] where they are mainly caused by the species: *L. major*, *L. infantum, L. donovani* or *L. tropica*. It is estimated that between 600 000 to 1 million new cases annually occur worldwide [2]. During the last years, migrations, climate change and variations, agricultural developments, conflicts constituted driving factors for emergence, changes in eco-epidemiology and geographical distribution of the parasites and diseases, and increased observation of atypical clinical presentations [2,3]. Refractory response to first line antimonial based treatment is also increasingly reported [4–8]. All these changing trends call for timely patient management and early alert on emerging trends.

Parasite detection and identification is a central issue to diagnosis and treatment decision, epidemiology, effective disease control and surveillance. Currently, Leishmaniases diagnosis focuses on parasite detection by microscopy, *in vitro* culture or PCR followed by sequencing or digestion by restriction enzymes of amplified products [9–11]. There is a need for fast, accurate, specific and sensitive methods for detection and identification of *Leishmania* species. High Resolution Melting analysis (HRM) lists among available used technologies for such purposes [12]. It is powerful, more rapid than classical PCR, sensitive and specific technology that uses the difference of melting curves due to sequence composition. It is sensitive even to a single nucleotide difference and is increasingly applied to identify or differentiate sequence genotypes for a range of needs such as the identification of recurrent genetic alterations useful for the diagnosis of the Philadelphia-negative Myeloproliferative Neoplasms (MPNs) [13], rapid genotyping tool for *Brucella* species [14], or simultaneous detection of five bacterial pathogens in urinary tract infections [15]. It was also described for the detection of infectious agents such as *Leishmania* targeting for instance *L. infantum* in different canine tissues [16] and for diagnosis of Leishmaniases in clinical samples [17]. In the case of infectious agents such as *Leishmania*, the success of HRM PCR assay depends on the choice of target [18]. So far, *Heat shock protein* 70 (*Hsp*70) [19–22], *Spliced Leader RNA* (*7SL RNA*) [17,22–25], Internal Transcribed Spacer 1 (ITS1) [26–28], a kinetoplast DNA (kDNA) minicircle [29,30] were used in HRM assays aiming at *Leishmania* species detection and identification. *Leishmania amino acid permease 3* coding sequences were also considered as promising targets for the development of HRM PCR assays, taking profit of this gene conservation and its levels of sequence homology across the different species [31], therefrom indicating relevance of unique copy coding sequences as *Leishmania* detection and identification targets for HRM.

*Strumpellin* is an ubiquitous protein present in cytosolic and endoplasmic reticulum cell compartments, encoded by a unique copy gene (gene *KIAA0196* in Human). It is a member of the multiproteic WASH regulatory complex implicated in the endosomal system [32–36], a highly conserved complex in different taxa that performs a crucial role in the structural integrity of endosomes and lysosomes [35]. The human *Strumpellin* belongs to a family of proteins which mutations have been shown to cause hereditary spastic paraplegia. Human *Strumpellin* protein consists of three parts: an N-terminal domain, a spectrin-like repeat domain in the central part that contains five repeats, and a C-terminal domain [33,35]. The spectrin repeat is a structural platform for cytoskeletal protein assemblies [32,33,37]. Bioinformatic analysis of the *Strumpellin* coding sequence has shown its structural and evolutionary conservation and its occurrence as a gene coding for a unique protein in a range of vertebrate organisms. It is also present in other taxa including insects, plants, nematodes, molds, and euglenozoans including *Leishmania* and *Trypanosoma* species, but it was absent in bacteria and fungi [33]. In *Leishmania*, the *Strumpellin* gene maps on chromosome 27, as a gene under a single copy (*LmjF.27.1660*; TriTrypDB). Phylogenetic analysis of the coding sequence in 100 organisms suggested its potential for taxonomy particularly for Euglenozoa [33].

In the present study, we aimed at investigating the potential use of the *Strumpellin* coding sequence as a target for the detection and identification of *Leishmania* species from Old World countries using HRM PCR technology, proving the concept for its usefulness for CL diagnosis.

## Methods

### Ethics statement

This study was approved by the “Comité d’Ethique Bio-Médicale de l’Institut Pasteur de Tunis” (Ref. 2016/13D/I/CIC, 2016/13D/I/CIC/amendement), in accordance with the Declaration of Helsinki. A written and verbal consent was obtained from the healthy donor (2018/07/I/LR11IPT04).

### Parasite strains

Forty-six cryopreserved and already typed *Leishmania* strains by isoenzyme analysis were used in this study. They were collected from field studies in Tunisia or were received from reference centers in Montpellier (J. P. Dedet) or London (D. A. Evans). They correspond to different *Leishmania* strains having diverse geographical origin, representing different species: *L. infantum* (N = 13)*, L. major* (N = 15)*, L. tropica* (N = 10)*, L. donovani* (N = 4)*, L. turanica* (N = 1)*, L. arabica* (N = 1) and *L. aethiopica* (N = 1), *L. tarentolae* (N = 1) as detailed in Table 1. Twenty-seven isolates (*L. infantum* (N = 5)*, L. major* (N = 19)*, L. tropica* (N = 3)) were further obtained from patients attending the parasitology department of Farhat Hached Hospital of Sousse, Tunisia, to confirm their CL diagnosis (Table 1). The parasites were isolated in coagulated rabbit serum media and mass grown in supplemented RPMI 1640 liquid cultures as described [38,39]. Total DNAs were purified using phenol-chloroform extraction and ethanol precipitation [40].

**Table 1:** *Leishmania* strains tested in this study and results obtained by the applied PCR assays.

Strain name	Code	Clinical form	Zymodeme	Species	Accession Numbers
MHOM/TN/80/IPT1^++^	IPT1	VL	MON-1	*L. infantum*	PQ470330; PQ495770
MHOM/TN/93/LV10	LV10	VL	MON-80	*L. infantum*	-
MHOM/TN/94/LV49^+^	LV49	VL	MON-24	*L. infantum*	PQ495771
MHOM/TN/94/LV50^+^	LV50	VL	MON-1	*L. infantum*	PQ495772
MHOM/TN/96/Drep08^+^	Dr8	CL	MON-1	*L. infantum*	PQ495773
MHOM/TN/97/Drep11^+^	Dr11	CL	MON-24	*L. infantum*	PQ495774
MHOM/TN/98/Drep15	Dr15	CL	MON-24	*L. infantum*	-
MHOM/DZ/01/LIPA1148	LIPA1148	VL	MON-1	*L. infantum*	-
MHOM/TN/97/Drep13^+^	D13	CL	MON-24	*L. infantum*	PQ495775
MHOM/TN/95/96/Drep05^+^	Dr5	CL	MON-1	*L. infantum*	PQ495776
MHOM/TN/95/LVGA^+^	LVGA	VL	-	*L. infantum*	PQ495777
MHOM/TN/92/LV08^+^	LV08	VL	-	*L. infantum*	PQ495778
MHOM/BR/74/PP75**^+^	PP75	VL	MON-1	*L. infantum*	PQ495779
MHOM/TN/11/EMPA4*	E4	VL	ND	*L. infantum*	-
MHOM/TN/11/EMPA8*	E8	VL	ND	*L. infantum*	-
MHOM/TN/12/EMPA38*	E38	VL	ND	*L. infantum*	-
MHOM/TN/12/EMPA39*	E39	VL	ND	*L. infantum*	-
MHOM/TN/12/EMPA40*	E40	VL	ND	*L. infantum*	-
MHOM/ET/67/HU3^++^	LEM698	VL	MON-18	*L. donovani*	PQ470329; PQ495780
MHOM/IN/00/DEVI^+^	LEM138	VL	MON-2	*L. donovani*	PQ495782
MHOM/SA/81/Jeddah-KA^+^	LEM536	VL	MON-2	*L. donovani*	PQ495781
MHOM/ET/72/GEBREI***	L1005	VL	MON-82	*L. donovani*	-
MPSA/TN/87/Ron102	Ron102	NA	MON-25	*L. major*	-
MPSA/TN/87/Ron150^+^	Ron150	NA	MON-25	*L. major*	PQ495756
MPSA/TN/87/Ron44^++^	Ron44	NA	MON-25	*L. major*	PQ470333
MPSA/TN/87/Ron101	Ron101	NA	MON-25	*L. major*	PQ495757
MPSA/TN/87/Ron155	Ron155	NA	MON-25	*L. major*	-
MPSA/TN/87/Ron99	Ron99	NA	MON-25	*L. major*	-
MRHO/SU/TN/59/P-strain	P-strain	NA	MON-4 LON-1	*L. major*	-
MMER/IN/73/GTBM	GTBM	NA	MON-23	*L. major*	-
MHOM/IL/80/Friedlin^+^	LEM3171	CL	MON-103	*L. major*	PQ495758
MHOM/IL/83/IL24^+^	IL24	CL	MON-66	*L. major*	PQ495759
MHOM/IL/83/IL32^+^	IL32	CL	MON-68	*L. major*	PQ495760
MHOM/DZ/89/LIPA1051	Lipa1051	CL	MON-25	*L. major*	-
MHOM/IL/67/Jericho II	JerichoII	CL	MON-26 LON-70	*L. major*	-
MHOM/IL/83/IL53	IL53	CL	MON-67	*L. major*	-
MHOM/SA/84/KFUH7532^+^	KFUH7532	CL	LON-65	*L. major*	PQ495761
MHOM/TN/06/NJ*	NJ	CL	ND	*L. major*	-
MHOM//TN/06/ Sgs*	Sgs	CL	ND	*L. major*	-
MHOM/TN/06/BNM*	BNM	CL	ND	*L. major*	-
MHOM/TN/06/KZ*	KZ	CL	ND	*L. major*	-
MHOM/TN/06/SF*	SF	CL	ND	*L. major*	-
MHOM/TN/06/GL*	GL	CL	ND	*L. major*	-
MHOM/TN/06/BaH*	BaH	CL	ND	*L. major*	-
MHOM/TN/05/BBC*	BBC	CL	ND	*L. major*	-
MHOM/TN/05/BAM*	BAM	CL	ND	*L. major*	-
MHOM/TN/06/BAR*	BAR	CL	ND	*L. major*	-
MHOM/TN/11/EMPA10*	E10	CL	ND	*L. major*	-
MHOM/TN/11/EMPA12*	E12	CL	ND	*L. major*	-
MHOM/TN/11/EMPA20*	E20	CL	ND	*L. major*	-
MHOM/ TN/11/EMPA21*	E21	CL	ND	*L. major*	-
MHOM/TN/ 11/EMPA22*	E22	CL	ND	*L. major*	-
MHOM/TN/11/EMPA23*	E23	CL	ND	*L. major*	-
MHOM/TN/11/EMPA24*	E24	CL	ND	*L. major*	-
MHOM/TN/11/EMPA25*	E25	CL	ND	*L. major*	-
MHOM/TN/11/EMPA29*	E29	CL	ND	*L. major*	-
MHOM/IL/00/Gabai 159^+^	Gabai159	CL	LON-9	*L. tropica*	PQ495766
MHOM/TN/06/AM*^+^	AM	CL	ND	*L. tropica*	PQ495767
MHOM/TN/06/CJ*^+^	CJ	CL	ND	*L. tropica*	PQ495768
MHOM/TN/06/Leep0920*^+^	L0920	CL	ND	*L. tropica*	PQ495769
MHOM/IQ/76/BAG9^++^	BAG9	CL	MON-53	*L. tropica*	PQ470334; PQ495763
MHOM/IQ/65/L75	L75	CL	MON-6	*L. tropica*	-
MHOM/IL/78/Rachnan	Rachnan	CL	MON-60	*L. tropica*	-
MHOM/AF/82/K001^+^	K001	CL	MON-58	*L. tropica*	PQ495764
MHOM/IQ/73/Bumm30^+^	Bumm30	CL	LON-17	*L. tropica*	PQ495765
MHOM/IQ/76/BAG17	BAG17	CL	LON-24	*L. tropica*	-
MHOM/SU/74/SAF-K27	SAF-K27	CL	MON-60	*L. tropica*	-
MHOM/IQ/73/A Sinai III	A sinai III	CL	LON-11	*L. tropica*	*-*
MHOM/GR/00/LA28^+^	LA28	CL	LON-16	*L. tropica*	PQ495762
MHOM/ET/72/L100^++^	L100	CL	MON-14	*L. aethiopica*	PQ470327; PQ495783
MPSA/SA/84/Jisha238^++^	Jisha238	-	LON-64	*L. arabica*	PQ470328; PQ495785
MRHO/SU/74/95-A^++^	95A	-	MON-65	*L. turanica*	PQ470332; PQ495784
IMIN/IT/86/MIN1^+^	Min I	NA	-	*L. tarentolae*	PQ470331

NA: Not Applicable, ND: Not Determined, -: Unknown, VL: Visceral Leishmaniasis, CL: Cutaneous Leishmaniasis, *: strains isolated from Tunisian patients attending the parasitology department of Farhat Hached Hospital of Sousse, Tunisia, **: *L. infantum* strain (also known as *L. chagasi*), ***: *L. donovani* strain (also known as *L. archibaldi*), ^+^: sequenced *Strumpellin* PCR product, ^++^: sequenced ITS1-PCR product (Accession numbers: PQ4703XX) and *Strumpellin* PCR products (Accession numbers: PQ4957XX).

Parasite species identification of the already identified laboratory strains and patients’ isolates was performed on the same DNA preparations using DNA probes [40,41], or using ITS1-PCR RFLP as described [42].

### Clinical samples

A total of 38 lesions aspirates were collected during 2010–2013 period from CL suspected patients referred to the Parasitology department of the Farhat Hached University Hospital in Sousse (Tunisia) to confirm diagnosis by smear examination. The remains of the aspirates used to make the smears on the slides were conserved in saline buffer at −20 °C and were anonymously used afterwards to extract the DNAs with the authorization of the ethics committee of the Institut Pasteur de Tunis. The DNAs were extracted from the frozen aspirates using the QIAamp DNA Mini Kit (Qiagen, Hilden, Germany) according to the manufacturer’s instructions (Table 2). Their *Leishmania* species identification was done applying the ITS1-PCR RFLP protocol [42].

**Table 2:** Clinical samples used in this study.

Patient	Year	Gender	Age	Number of skin lesions	Geographical origin
Pa1	2011	F	12	2	Sousse
Pa2	2011	F	12	5	Mahdia
Pa3	2011	F	28	3	Mahdia
Pa4	2011	M	63	2	Sousse
Pa5*	2011	M	50	1	Sousse
Pa6	2011	F	23	3	Mahdia
Pa7	2011	M	32	1	Kasserine
Pa8	2011	F	7	3	Sidi Bouzid
Pa9*	2012	F	70	1	Kairouan
Pa10	2013	F	39	1	Jendouba
Pa11	2011	F	25	7	Sidi Bouzid
Pa12	2011	M	81	1	Kasserine
Pa13	2011	F	18	2	Kasserine
Pa14	2011	F	2	1	Sousse
Pa15	2011	F	33	1	Sidi Bouzid
Pa16	2011	M	14	2	Kairouan
Pa17	2011	M	37	1	Mahdia
Pa18	2011	F	25	2	Seliana
Pa19	2011	F	25	1	Kairouan
Pa20*	2010	F	36	1	Sousse
Pa21	2010	M	4	1	Jendouba
Pa22	2010	F	56	1	Seliana
Pa23	2011	F	51	1	Sousse
Pa24*	2011	M	35	1	Mahdia
Pa25	2011	M	34	6	Sidi Bouzid
Pa26	2012	F	3	1	Sousse
Pa27	2010	F	43	1	Kairouan
Pa28	2010	F	52	1	Gafsa
Pa29	2010	M	71	1	Monastir
Pa30	2010	F	31	5	Mahdia
Pa31	2011	M	48	4	Sidi Bouzid
Pa32	2011	F	3	1	Sidi Bouzid
Pa33	2010	F	36	1	Kasserine
Pa34	2010	F	35	2	Kairouan
Pa35	2010	F	22	3	Sousse
Pa36	2010	M	13	1	Mahdia
Pa37	2011	F	39	1	Kasserine
Pa38	2010	M	7	6	Sousse

Pa: Patient, F: Female, M: Male, *: sequenced *Strumpellin* PCR product, Accession numbers: Pa5 (PQ495786), Pa9 (PQ495787), Pa20 (PQ495788), Pa24 (PQ495789).

Human DNA extracted from the blood of a healthy donor was used to control the specificity of the primers in the HRM PCR analysis.

### Direct examination

To confirm suspected cases of CL, dermal smears were fixed with methanol and stained with Giemsa. Then, the slides were examined at the x100 objective using immersion oil to detect *Leishmania* amastigotes.

### *In silico* and evolution studies of the *Strumpellin* gene

To confirm the potential use of the *Strumpellin* protein as a diagnostic target, we first confirmed the annotation of the gene by comparing the protein encoded by *LmjF.27.1660* gene to the human *Strumpellin* protein and analysing its domains using Pfam and InterPro software.

To evaluate the protein sequence conservation in the human and the *L. major Strumpellin* encoded by *LmjF.27.1660*, we compared the amino acid composition using Geneious V3.6.2 program and evaluated the presence of conserved motifs using MEME software (version 5.5.4). It was used by applying the following data submission parameters: 10 motifs, number of residues in each motif varies between 6 and 15, and the other settings as default.

We then conducted a bioinformatic analysis of the *Strumpellin* coding sequences using available Trypanosomatidae genomes on the TriTrypDB database (Release 56, 15 Feb 2022). For this purpose, a BLAST analysis using *LmjF.27.1660* as bait identified 50 sequences referring to *Trypanosomatidae* species. Based on total scores, e-value (0.00 E^+00^), query cover (44%–100%) and percent identity (83%–100%), 29 sequences were retained corresponding to 19 species thereof 15 *Leishmania* species (N = 25), 2 *Leptomonas* species (N = 2), 1 *Endotrypanum* species (N = 1) and 1 *Crithidia* species (N = 1). The Geneious v3.6.2 program was used to estimate the average sequence composition and the average percentage of pairwise similarity over the alignment of the 29 *Strumpellin* sequences selected, as recommended by the software. For the phylogenetic analysis of this gene, the MEGA software (Version 11) was used through the Bayesian information criterion. Their phylogeny was computed with Tamura 3-parameter method [43,44] with Gamma distribution variation rate [45]. Trees were constructed with Maximum likelihood [46], Neighbor Joining [47], UPGMA [48], and Minimum Evolution [49] using 1000 bootstrap resampling method [50].

Two computer methods available on MEGA software (Version 11), pairwise distance and Codon-based Z-test of selection, were used to assess the selection pressure of the entire *Strumpellin* encoding gene. We measured the difference between the synonymous and non-synonymous substitutions (dN-dS). The pairwise method used the Nei-Gojobori model and Gamma distribution with bootstrap method for variance estimation (bootstrap = 1000). The Codon-based Z-test of selection method was estimated using purifying selection test hypothesis (HA: dN < dS), the Nei-Gojobori model and bootstrap method for variance estimation (bootstrap = 1000). We considered 25 *Leishmania* genus sequences. They include 16 *L. (Leishmania)* (14 Old World, 2 New World), 2 *L.* (*Sauroleishmania)*, 5 *L. (Viannia)* and 2 *L. (Mundinia)* subgenera sequences.

We also analyzed the selection pressure of the selected target used for HRM PCR as described previously. We considered 33 sequences belonging to various *Leishmania* subgenera: *L. (Leishmania)* (N = 16), *L.* (*Viannia)* (N = 5), *L. (Mundinia)* (N = 6) and *L.* (*Sauroleishmania)* (N = 2), and to *Endotrypanum* (N = 1), *Crithidia* (N = 1) and *Leptomonas* (N = 2) species.

### Primers design

A comparative study of different *Leishmania* coding sequences for the *Strumpellin* protein was performed to identify species-specific polymorphisms that could be useful to develop an HRM PCR method. The *Leishmania* sequences that were retrieved from TriTrypDB (Release 56, 15 Feb 2022) were aligned using Geneious software (Geneious v.3.6.2). In addition to criteria described for the selection of PCR primers [51], several others were considered to select for efficient HRM PCR primers [52] that would be in regions with high homology flanked by regions containing low intraspecific and high interspecific variability, generating small length amplicons (<300bp). Two sets of PCR primer pairs were selected manually. The first pair, KF4/KR4 (KF4: 5’-TCACTACTACGGCGGGTACA-3’, KR4: 5’-ATGCGCTGCAAGTGATAGGT-3’), was designed using HRM criteria in a conserved region flanking species-specific polymorphisms in a multiple sequence alignment, and retained for further investigation. “e-PCR” confirmed that the primers only match with unique sites within the gene to yield a fragment of the expected size in *L. major*. The second pair, MI5032F/R (MI5032F: 5’-AAGGAGTTGGTGGAGAAGCA-3’; MI5032R: 5’-AGCGTCAGAAGCTCATCGTT-3’), was designed to set up a conventional PCR that amplifies a fragment containing the KF4/KR4 target, for purposes of sequence analyses.

To confirm the specificity of the selected priming sites of the KF4/KR4 to a group of *Leishmania* species, the sequence alignment considered a total of 33 sequences belonging to various *Leishmania* subgenera: *L. (Leishmania)* (N = 16), *L.* (*Viannia)* (N = 5), *L.* (*Mundinia)* (N = 6) and *L*. *(Sauroleishmania)* (N = 2), and to *Endotrypanum* (N = 1), *Crithidia* (N = 1) and *Leptomonas* (N = 2) species.

### PCR assays

#### ITS1-PCR RFLP.

The ITS1-PCR RFLP using the primers LITSR: 5’- CTGGATCATTTTCCGATG-3’ and L5.8S: 5’-TGATACCACTTATCGCACTT-3’ was carried out as described by Schonian et al., 2003 [42]. The positive ITS1-PCR products were digested with *HaeIII* (Vivantis, Selangor Darul Ehsan, Malaysia) and the restriction fragments were analyzed by electrophoresis on 3% agarose gels. The RFLP profiles identify the species as described [42]. This assay was used to reconfirm or identify the species assignment of strains and isolates of this study and the infecting species of the cutaneous samples.

To confirm RFLP patterns, the ITS1-PCR products of a selection of representative *Leishmania* species (*L. major, L. infantum, L. tropica, L. donovani, L. turanica, L. aethiopica, L. arabica, L. tarentolae*) DNAs were sequenced and analyzed by BLAST. *In silico HaeIII* digestion of the obtained sequences was conducted using SnapGene version 7.2.1. The sequences were deposited in NCBI under the accession numbers provided in Table 1.

#### B-globin PCR assay.

A B-globin PCR assay, targeting the human *beta-globin* gene, was performed to confirm the presence/absence of PCR inhibitors or/and check quality of DNA, as described [53].

#### HRM PCR assay.

The primer’s pair, KF4/KR4, was used to amplify a 120bp fragment in a Light Cycler 480 Instrument (Roche, France). Optimal amplification reaction conditions using the Light Cycler 480 High Resolution Melting Master Mix (Roche, France) were set for the reaction to be performed in a total volume of 10 µL containing 20 ng of DNA, 0.2 µM of each primer, 2 mM MgCl_2_, 1x PCR master Mix and ultra-pure grade PCR water (Roche, France). Reaction cycles conditions were as follows: an initial denaturation for 5 min at 95 °C followed by 40 cycles of denaturation for 10 sec at 95 °C, annealing for 10 sec at 63 °C and extension for 15 sec at 72 °C. This was followed by a melting step from 55 °C to 95 °C, using a ramping of 0.02 °C/sec. Two negative controls without DNA template were included in each experiment. These conditions were set on representative *Leishmania* DNAs, by evaluating different concentrations of MgCl_2_ and different total reaction volumes. HRM data were analyzed using the Gene Scanning method/Tm calling/Fit points available on Light Cycler 480 software, supplied with the machine.

### PCR assay for sequence analyses

Another PCR assay was developed using MI5032F/R primers that are flanking the fragment amplified by KF4/KR4 HRM PCR. This primer set was used to compare the HRM PCR assay results and the nucleotide sequence composition of the amplified fragments. This PCR was performed in a total volume of 25 µL, containing 0.2 µM of each primer MI5032F/R, 0.2 mM of each dNTPs, 1.5 mM MgCl_2_, 0.25 U of Go Taq DNA polymerase (Promega, Lyon, France) and 20 ng of *Leishmania* DNA. Amplification cycles conditions were as follows: an initial denaturation for 5 min at 95 °C was followed by 30 times repeated cycles of (denaturation for 1 min at 95 °C, annealing for 1 min at 58 °C, extension for 1 min at 72 °C) and then a final extension for 5 min at 72 °C. A 452bp fragment was observed with all *Leishmania* DNAs on 1.5% agarose gel.

The PCR products were sequenced using ABI PRISM 3130 Genetic Analyzer (Foster City, CA, USA). PCR products were purified using EXO-SAP method (Thermo scientific) and sequenced using the kit BigDye Terminator v3.1 (Applied Biosystems, USA). DNA Baser Assembler v5.15.0 was used to visualize chromatograms, correct sequences manually and assemble contigs. The contigs were blasted for homology search in TritrypDB and analyzed using Geneious v.3.6.2 program to identify SNPs. The obtained sequences were deposited in NCBI under the accession numbers provided in (Tables 1 and 2).

### Statistical analysis

Sensitivity, specificity, Positive Predictive Values (PPV) and Negative Predictive Values (NPV) (95% CI) were determined, considering Direct examination as the gold standard method. *Kappa* value was also calculated to evaluate the degree of agreement between Direct Examination, ITS1-PCR and HRM PCR according to Hassine et al. (2022) [54]. The *kappa* value interpretation is suggested as follows: values ≥0.81 as excellent agreement, 0.61–0.80 as good, 0.41–0.60 as moderate, 0.21–0.40 as fair, 0.01–0.020 as none to slight, <0.0 no agreement [55].

Fisher’s exact test statistics was calculated using the Social Science Statistics website (https://www.socscistatistics.com/tests/) to evaluate the correlation between HRM PCR and ITS1-PCR. The result is considered significant at *p* value <0.05.

## Results

### *In silico* analyses of the *Strumpellin* protein and coding sequences confirmed its potential for *Leishmania* identification

The *Strumpellin* protein in humans has different aliases (WASHC5, WASH complex subunit 5, WASH complex subunit strumpellin, etc (https://www.genecards.org/cgi-bin/carddisp.pl?gene=WASHC5). In *Leishmania*, there is little information about the protein. The annotation of the predicted *L. major* protein as *Strumpellin* was confirmed by its descriptive analysis using Pfam and InterPro software. The analysis results assigned the *Leishmania* protein to the *Strumpellin* protein (PF10266; IPR019393).

**Fig 1:**
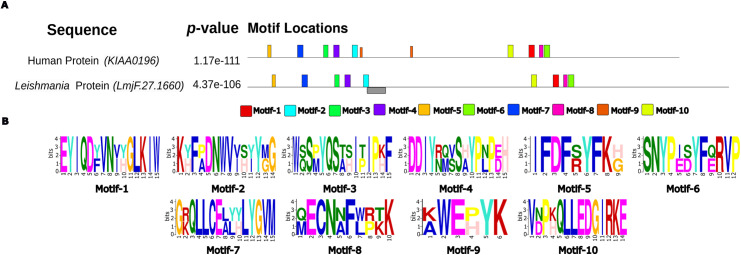
Conserved motif analysis of Human and *Leishmania Strumpellin* proteins using MEME suite. **(A)** Schematic diagram of amino acid motifs of *Strumpellin* proteins. Motif analysis was performed by MEME (Multiple Em for Motif Elicitation, software version 5.5.4, http://meme-suite.org) using a maximum number of 10 motifs without restriction of the number of site distribution and a minimum and a maximum width comprised between 6 and 15 residues. The rest of parameters was kept as default. Black lines correspond to the *Strumpellin* Proteins. Colored boxes represented the different motifs and their position in each protein. Grey box correspond to the selected target (320–359) used for the development of the HRM PCR. **(B)** Conserved consensus motifs identified by MEME suite are Motif-1 (EYIQDFVNIHGLKIW), Motif-2 (KHFPDNWVIHIYGG), Motif-3 (WQSPYQSAHIPIPKF), Motif-4 (DDIYRQVSAYPNPEH), Motif-5 (IFDFRYFKG), Motif-6 (SNYPEDYFZRVP), Motif-7 (GKQLLCEALHLYGVM), Motif-8 (QECNNFWRKK), Motif-9 (KWEPYK), Motif-10 (VDPKQLLEDGIRKE).

A comparative analysis of the human *Strumpellin* (*KIAA0196*) and the predicted *Leishmania major* protein encoded by *LmjF.27.1660*, to evaluate the conservation level of the protein sequences, revealed their different amino acid sequence composition with average 32% of pairwise similarity and 35.7% of identical sites. In humans, the protein annotation identifies a large central Spectrin repeat region (known to contain 5 repeats [33]), N-terminal and C-terminal regions [33]; in *Leishmania*, the interPro program predicted the presence of five domains (cytoplasmic domain, coil, disorder prediction, transmembrane region and non-cytoplasmic domain) on the *L. major* protein (S1 Fig) but we did not observe internal repeats. Therefore, we used MEME to model and compare the proteins’ primary sequence by their amino-acid motifs. The 2 proteins shared 10 conserved motifs with width varying between 6 and 15 residues that have similar organization and respective spacing. All this confirmed *Strumpellin* annotation of the predicted *Leishmania* protein and revealed a close yet different architectural resemblance to human *Strumpellin*, while also highlighting some of its unique features (Fig 1).

Then a bioinformatic analysis of the *Strumpellin* coding sequence was conducted to confirm the taxonomical potential of the gene. Search in TriTrypDB database of the protein coding genes in the different available genomes showed its conservation as a unique copy gene and its conserved synteny within the Trypanosomatidae family, including different *Leishmania* subgenera *L.* (*Leishmania), L. (Viannia), L. (Sauroleishmania), L. (Mundinia)* and other Kinetoplastid species: *Crithidia*, *Endotrypanum* and *Leptomonas* (S2 Fig). It is mapped on chromosome 27 in *Leishmania* under one copy on the haploid genome. Twenty-nine sequences corresponding to 19 species (15 *Leishmania* species, 2 *Leptomonas* species, 1 *Endotrypanum* species and 1 *Crithidia* species) were retained through the BLAST analysis of the *Strumpellin* coding sequence using Trypanosomatidae genomes, according to the total scores, e-value, query coverage and percent identity. The multiple sequence alignment of the 29 sequences performed by Geneious program showed average pairwise similarity of the *Strumpellin* gene estimated to 86.3% and 44.2% identical sites. The sequence analysis of the entire gene showed the average nucleotide frequencies of A (18.6%), C (29.8%), G (31.5%), T (20.1%) and its GC content (61.3%) (S3 Fig).

**Fig 2:**
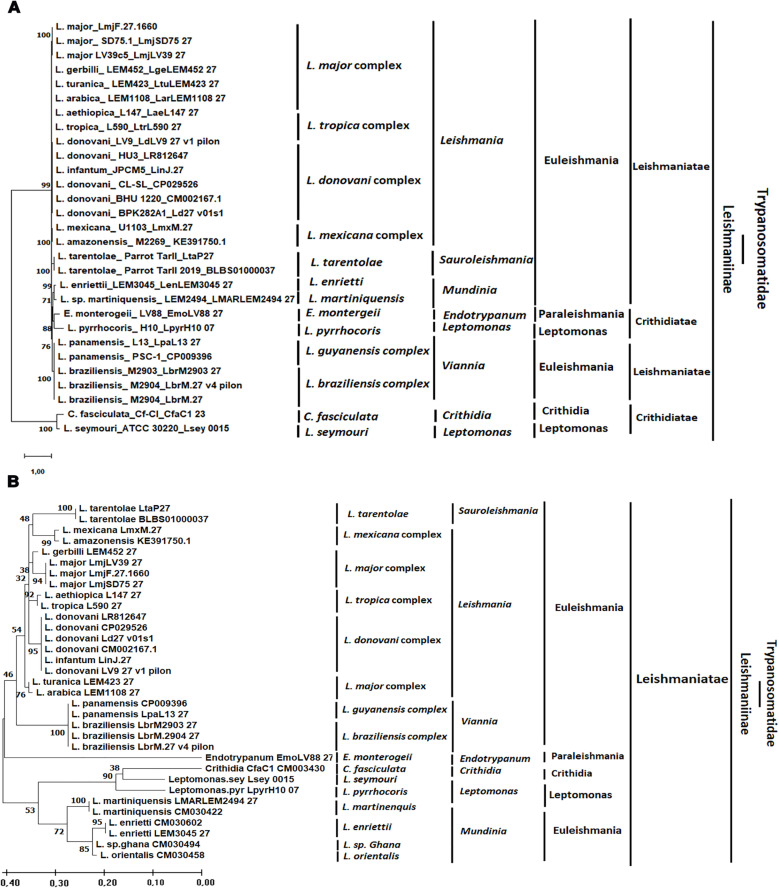
Evolutionary analysis of the *Strumpellin* coding sequences by Maximum Likelihood method. **(A)** Analysis of the studied entire coding sequences. The evolutionary history was inferred by using the Maximum Likelihood method and Tamura 3-parameter model [43]. The tree with the highest log likelihood (−27440.00) is shown. The percentage of trees in which the associated taxa clustered together is shown next to the branches. Initial tree(s) for the heuristic search were obtained automatically by applying Neighbor-Join and BioNJ algorithms to a matrix of pairwise distances estimated using the Tamura 3 parameter model, and then selecting the topology with superior log likelihood value. A discrete Gamma distribution was used to model evolutionary rate differences among sites (5 categories (+G, parameter = 3.2266)). The tree is drawn to scale, with branch lengths measured in the number of substitutions per site. This analysis involved 29 nucleotide sequences. Codon positions included were 1st + 2nd + 3rd + Noncoding. There were a total of 3960 positions in the final dataset. Evolutionary analyses were conducted in MEGA11 [44]. The classification was made based on the literature review of Trypanosomatidae and *Leishmania* classification [56–59]: Trypanosomaidae (Family), Leishmaniinae (Subfamily), Leishmaniatae (Infrafamily). **(B)** Analysis of the HRM PCR target. The evolutionary history was inferred by using the Maximum Likelihood method and Tamura 3-parameter model [43]. The tree with the highest log likelihood (−796.18) is shown. The percentage of trees in which the associated taxa clustered together is shown next to the branches. Initial tree(s) for the heuristic search were obtained automatically by applying Neighbor-Join and BioNJ algorithms to a matrix of pairwise distances estimated using the Tamura 3 parameter model, and then selecting the topology with superior log likelihood value. A discrete Gamma distribution was used to model evolutionary rate differences among sites (5 categories (+G, parameter = 1.0234)). The tree is drawn to scale, with branch lengths measured in the number of substitutions per site. This analysis involved 33 nucleotide sequences. Codon positions included were 1^st^ + 2^nd^ + 3^rd^ + Noncoding. There were a total of 120 positions in the final dataset. Evolutionary analyses were conducted in MEGA11 [44].

Phylogenetic analysis using Maximum Likelihood, UPGMA, Neighbor Joining and Minimum Evolutionary methods were used to generate phylogenetic trees showing similar tree topologies with the different computational methods. The analyzed sequences are organized in clusters in congruence with the updated classification of *Leishmania* species reported by Akhoundi et al. (2017) [56]. The Tree topology follows the classification of the Trypanosomatidae family into subfamily (Leishmaniinae) and infrafamily (Leishmaniatae) [57–59] (Fig 2A).

Then we measured the selection pressure on the gene across the different subgenera and species to confirm their taxonomical relevance using two computational methods: pairwise distance (dN-dS), and Codon-based Z-test of selection under the test hypothesis of purifying selection (dN < dS) where the hypothesis is maintained when *p* value is less than 0.05. In case of 25 *Leishmania* sequences corresponding to 15 *Leishmania* species belonging to different subgenera the dN-dS equals −0.2378 with the first method and dS-dN = 25.50 with *p* = 0.00 in the second method indicating that the genes are under a negative selection pressure, and so that this gene is particularly relevant for these organisms. This was further confirmed for *Strumpellin* encoding genes belonging to 8 Old World *Leishmania* (*Leishmania*) species and 1 *L. (Sauroleishmania)* species: *L. donovani, L. infantum, L. tropica, L. aethiopica, L. turanica, L. arabica, L. major, L. gerbilli, L. tarentolae*. Using the two computational methods, the pairwise distance dN-dS equals −0.1081, and with the Codon-based Z-test of selection with test hypothesis of purifying selection (dN < dS), dS-dN was found 16.11 with *p* = 0.00 (Table 3). This highlighted the importance of the gene in *Leishmania* parasites biology.

**Table 3:** Selection pressure analysis of the *Strumpellin* gene and the selected HRM target.

	Subgenera	Species	Sequences	Z-score (dN < dS)*	Distance (dN-dS)**
***Strumpellin* gene** **(Old and New World species)**	*L. (Leishmania)*	10	16	dS-dN = 25.50, *p* = 0.00 (<0.05)	−0.2378
	*L. (Viannia)*	2	5		
	*L. (Sauroleishmania)*	1	2		
	*L. (Mundinia)*	2	2		
	**Total**	**15**	**25**		
***Strumpellin* gene** **(Old World species)**	*L. (Leishmania)*	8	14	dS-dN = 16.11, *p* = 0.00 (<0.05)	−0.1081
	*L. (Sauroleishmania)*	2	2		
	**Total**	**10**	**16**		
**HRM Target on the *Strumpellin* gene** **(Euleishmania, Paraleishmania, Crithidia, Leptomonas)**	*L. (Leishmania)*	10	16	dS-dN = −2.35; *p* = 1.00 (>0.05)	2.9242
	*L. (Sauroleishmania)*	1	2		
	*L. (Viannia)*	2	5		
	*L. (Mundinia)*	4	6		
	*Endotrypanum*	1	1		
	*Crithidia*	1	1		
	*Leptomonas*	2	2		
	**Total**	**21**	**33**		

* Purifying selection (dN < dS): Values of P less than 0.05 are considered significant at the 5% level and are highlighted; ** (dN-dS): the difference between the nonsynonymous and synonymous distances per site from between sequences are shown; dS and dN are the numbers of synonymous and nonsynonymous substitutions per site, respectively; *L.* (*Leishmania)* species: *L. donovani*, *L. infantum*, *L. tropica*, *L. aethiopica*, *L. turanica*, *L. arabica*, *L. major*, *L. gerbilli*, *L. mexicana*, *L. amazoniensis*; *L. (Viannia)* species: *L. braziliensis*, *L. panamensis*; *L.* (*Sauroleishmania)* species: *L. tarentolae*; *L. (Mundinia)* species: *L. enriettii*, *L. martiniquensis*.

### Design of an HRM PCR assay

To design amplification primers, a multiple sequence alignment of the entire *Strumpellin* coding sequence (CDS) was carried out considering the 16 different sequences deposited for *Leishmania* species and strains belonging to the 8 Old World *Leishmania* species and 1 *L.* (*Sauroleishmania): L. major, L. infantum, L. donovani, L. tropica, L. aethiopica, L. arabica, L. turanica, L. gerbilli, L. tarentolae*. Different polymorphic sites were identified in the entire *Strumpellin* CDS within *Leishmania* species and between them, which we considered to design an HRM PCR assay. For this purpose, we chose a short DNA region within the *Strumpellin* gene (683756–683875bp on chromosome 27) having limited inter- and intra- species polymorphisms to obtain simple and differential melting curves as recommended [26,60] that could also be observed consistently at the taxonomic level. A first primer pair, KF4/KR4 (960–979bp; 1060–1079bp on the gene), was designed to target this gene fragment, each oligonucleotide matching a conserved region, to allow the amplification of 120bp fragment for the subgenus *Leishmania* species belonging to the Old World; these priming sites are flanking 17 variable sites that could distinguish *Leishmania* species or group of species.

**Fig 3:**
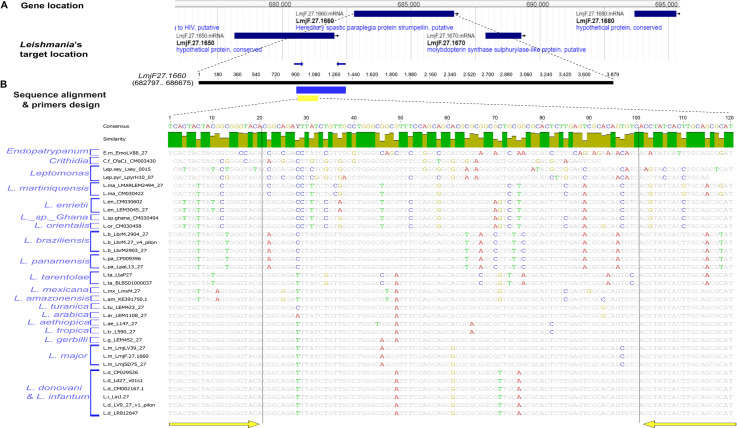
Design of HRM PCR primers targeting the *Strumpellin* protein coding sequence. **(A)** The *Strumpellin* gene is located on chromosome 27 at position 682797 - 686675 in *L. major* genome. **(B)** The target was blasted using TriTrypDB, sequences corresponding to *L. major* (Friedlin, LV39c5, SD75.1)*, L. infantum* (JPMC5), *L. donovani* (BHU 1220, BPK282A1, CL-SL, HU3, LV9), *L. tropica* (L590), *L. aethiopica* (L147), *L. arabica* (LEM1108), *L. turanica* (LEM423), *L. gerbilli* (LEM452), *L. mexicana* (LmxM.27), *L. amazonensis* (KE391750.1), *L. tarentolae* (BLBS01000037, LtaP27), *L. braziliensis* (LbrM2903_27, LbrM.27, LbrM.27_v4_pilon), *L. panamensis* (LpaM13_27, CP009396), *L. enrietii* (LenLEM3045_27, CM030602), *L. martiniquensis* (LMARLEM2494_27, CM030422), *L. sp. Ghana* (CM0330494), *L. orientalis* (CM0330458), *Endotrypanum* (EmoLV88_27), *Crithidia* (CfaC1_23), *Leptomonas* (Lsey_0015, LpyrH10_07) were retrieved and aligned using Geneious software. A PCR primer pair MI5032F/R (blue arrows) was designed on conserved regions to develop a conventional PCR. A second primer pair KF4/KR4 (yellow arrows) was designed within this fragment to develop an HRM PCR assay for the detection and identification of Old World *Leishmania* species.

To confirm the specificity of the selected priming sites, we considered a multiple sequence alignment of 33 sequences belonging to *L. (Leishmania)* (N = 16), *L. (Viannia)* (N = 5), *L.* (*Mundinia)* (N = 6) and *L.* (*Sauroleishmania)* (N = 2) subgenera and other species: *Endotrypanum* (N = 1), *Crithidia* (N = 1) and *Leptomonas* (N = 2) species. The sequence alignment confirmed the conservation of the priming sites within the Old World *Leishmania* species (Fig 3). With primer BLAST, we have also observed only one fragment matching with the 2 primers. The comparative analysis of the fragment targeted by this primer set across the panel of sequences in Geneious v3.6.2 program showed average 85.1% of pairwise similarity and 46.7% of identical sites. The average nucleotide frequencies were also estimated for A (18%), C (32.4%), G (28%), T (21.5%) and GC content (60.4%).

Phylogenetic analysis and the selection pressure on this fragment were also studied. In the 33 sequences belonging to *L. (Leishmania)* (N = 16), *L. (Viannia)* (N = 5), *L. (Mundinia)* (N = 6) and *L.* (*Sauroleishmania)* (N = 2) subgenera and to *Endotrypanum* (N = 1), *Crithidia* (N = 1) and *Leptomonas* (N = 2) species, we noted a similar tree topology to the one observed with the alignment of the entire gene. Additionally, the analyzed sequences were organized in clusters that matched the classification within the Trypanosomatidae family (Fig 2B) [56–59].

Afterwards, the selection pressure of the amplified sequence (KF4/KR4) was evaluated using the two computational methods: pairwise distance (dN-dS = 2.9242) and Codon-based Z-test of selection, test hypothesis was purifying selection (dN < dS) dS-dN = −2.35; *p* = 1.00; the hypothesis is rejected when *p* value is more than 0.05; these results highlighted that the target is under positive selection (Table 3).

The negative selection pressure on the *Strumpellin* gene and the positive one on its HRM PCR target, besides the conserved tree topology, confirmed the relevance of this gene and the selected target for taxonomical purposes.

To further investigate the KF4/KR4 region for potential occurrence of polymorphisms in the KF4/KR4 priming sites or within the fragment, a second primers’ pair, MI5032F/R, matching conserved regions upstream and downstream the KF4/KR4 primers, respectively (Fig 3), was designed to set up a conventional PCR to amplify a 452bp fragment for sequence analyses. For this validity check, we sequenced 30 MI5032F/R PCR products corresponding to different *Leishmania* species and strains having different geographical, host or clinical origins: *L.* major (N = 6), *L. infantum* (N = 10), *L. tropica* (N = 8), *L. donovani* (N = 3), *L. turanica* (N = 1), *L. arabica* (N = 1), *L. aethiopica* (N = 1) (Table 1). We aligned these sequences to the ones retrieved from the databases using Geneious software, which confirmed the conservation of the KF4/KR4 priming sites in Old World *Leishmania* species (S4 Fig). Only two variable sites were detected on these primer sites in the *L. tarentolae* DNA sequence.

The 17 polymorphic sites, which altogether differentiated between the following taxa: *L. donovani complex, L. major, L. tropica, L. turanica, L. arabica, L. aethiopica* were confirmed. No other polymorphic sites were observed. In total, 10 sequence types were observed: One was specific to the *L. donovani* complex (G1); the tested *L. infantum* and *L. donovani* DNAs presented the same sequence of the amplified sequence). Specific genotypes were observed each for *L. major* strains (G2), *L. aethiopica* (G6), *L. arabica* (G7), *L. turanica* (G8), *L. gerbilli* (G9) and *L. tarentolae* (G10) species. Three genotypes were observed for *L. tropica* parasites (G3, G4, G5) which were due to a single polymorphism within the species noted on the fragment’s position 45 (Table 4). In the case of BAG9, K001 and Bumm30 *L. tropica* strains (G4), a clear heterozygous SNP was seen with 2 overlapping peaks, one reading a C while the other an A. In the other cases, the *L. tropica* strains had a clear peak corresponding to an A (LA28) (G5) or a C (AM, CJ, L0920, Gabai159) (G3) indicating the position is homozygous. This nucleotide position thus differentiates the three genotypes of *L. tropica*. Likewise, the tested *L. aethiopica* (G6) and *L. tropica* strains differ by this same position with a T in the first case instead of an A and/or a C in the second. *L. arabica* (G7) and *L. turanica* (G8) also differ by one SNP in position 28 (A *vs* C).

**Table 4:** Sequence analysis of the HRM PCR target in representative DNAs of different *Leishmania* species.

Species	Origin	Number of strains	Gene ID/Accession numbers	Representative strains of each genotype	Polymorphic sites																			Genotype
					10	28	34	45	46	49	61	65	67	71	73	75	76	82	93	94	97	99	115	
*L. donovani complex*	TriTrypDB	6	LdBPK.27.2.001560	LV9	C	T	T	C	G	A	G	G	G	T	G	A	G	T	A	C	T	T	G	1
	Local	13	PQ495770	IPT1																				
*L. major*	TriTrypDB	3	LMJSD75_270023100	SD75.1	-	-	-	-	A	G	-	-	-	C	-	G	-	-	-	-	C	-	-	2
	Local	6	PQ495756	Ron150																				
*L. tropica*	TriTrypDB	1	LTRL590_270023000	L590	-	-	-	-	-	-	A	A	-	C	-	G	-	C	-	-	-	-	-	3
	Local	4	PQ495766	Gabai159																				
		3	PQ495765	Bumm30	-	-		C/A	-	-	A	A	-	C	-	G	-	C	-	-	-	-	-	4
		1	PQ495762	LA28	-	-	-	A	-	-	A	A	-	C	-	G	-	C	-	-	-	-	-	5
*L. aethiopica*	TriTrypDB	1	LAEL147_000471700	L147	-	-	-	T	-	-	A	A	-	C	-	G	-	C	-	-	-	-	-	6
	Local	1	PQ495783	L100																				
*L. arabica*	TriTrypDB	1	LARLEM1108_270023300	LEM1108	-	A	-	-	-	-	A	-	-	C	-	G	-	-	G	-	-	-	-	7
	Local	1	PQ495785	Jisha238																				
*L. turanica*	TriTrypDB	1	LTULEM423_270022900	LEM423	-	C	-	-	-	-	A	-	-	C	-	G	-	-	G	-	-	-	-	8
	Local	1	PQ495784	95A																				
*L. gerbilli*	TriTrypDB	1	LGELEM452_270023400	LEM452	-	-	-	-	A	-	A	-	-	C	-	G	-	-	-	-	-	-	-	9
*L. tarentolae*	TriTrypDB	1	LtaP27.1730	Parrot Tar II 2019	T	-	G	-	C	-	A	-	C	G	T	G	A	-	-	A	-	C	A	10

Gene IDs were retrieved from TriTrypDB.

All SNPs observed on the sequences retrieved from the databases were also seen by sequence analyses on the 30 DNAs listed in Table 4. All these results confirmed the relevance of the selected fragment for the design of an HRM PCR assay.

### Development of an HRM PCR assay targeting *Leishmania Strumpellin* gene

**Fig 4:**
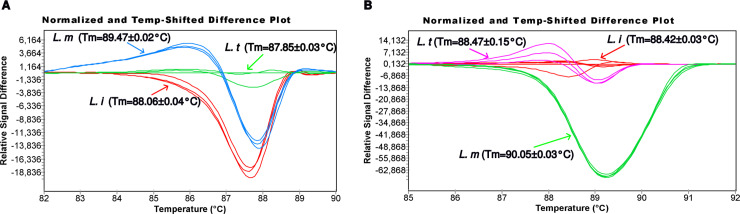
Development of a sensitive HRM PCR analysis targeting the *Strumpellin* protein coding sequence. **(A)** An HRM PCR assay was set up to be performed on a Light Cycler 480 Instrument. The analyses using the Gene scanning software allowed determining melting curves that are here represented in blue, red and green curves for the *L. major* (LEM3171: Tm = 89.46 °C, Ron44: Tm = 89.45 °C, Ron102: Tm = 89.50 °C), *L. infantum* (IPT1: Tm = 88.02 °C, LV08: Tm = 88.08 °C, Dr5: Tm = 88.09 °C) and *L. tropica* (SAF-K27: Tm = 87.87 °C, BAG9: Tm = 87.88 °C, K001: Tm = 87.82 °C) strains, respectively. The HRM PCR using KF4/KR4 detected and easily identified the 3 *Leishmania* species DNAs according to their melting curves. **(B)** The analytical limit of detection of the *Strumpellin* HRM PCR assay was measured by 8 times 10-fold dilution (20 ng to 2 fg) of *L. infantum* (IPT1), *L. major* (LEM3171) and *L. tropica* (BAG9) DNAs. The mean Tm were determined using the Tm calling software. Tm values corresponding to the *Leishmania* DNA amounts are described in S1 Table. The run was tested in triplicate. The HRM PCR was able to detect *Leishmania* DNA down to 2 pg with the three *Leishmania* species. The analyses using the Gene scanning software showed the conservation of the melting curves and the ability to differentiate between the 3 *Leishmania* species represented in red, pink and green curves for the *L. infantum* (IPT1), *L. tropica* (BAG9) and *L. major* (LEM3171), respectively.

To confirm the potential of the KF4/KR4 HRM PCR primers, we have set up an HRM assay and tested the selected reaction conditions for consistent profiles within a given species. The HRM assay conditions were thus applied to 9 representative strains corresponding to *L. major* (Ron102, LEM3171, Ron44), *L. infantum* (IPT1, LV08, Dr5) and *L. tropica* (K001, SAF-K27, BAG9) species. Three different melting curves were obtained for the three *Leishmania* species as illustrated in Fig 4A. Light cycler 480 software-based analyses determined diverse melting curves and temperatures. Each melting curve corresponded to one *Leishmania* species; the same one was obtained for all strains tested of each species.

Furthermore, the melting temperature was evaluated with the different strains tested of the *L. major, L. infantum* and *L. tropica* species. The mean Tm values of the different *Leishmania* species, *L. major, L. infantum* and *L. tropica,* were 89.47 ± 0.02 °C, 88.06 ± 0.04 °C and 87.85 ± 0.03 °C, respectively. These results showed a consistent melting peak for each *Leishmania* species. So, the developed HRM PCR assay was able to detect and clearly identify the 3 *Leishmania* species according to their conserved melting curves and melting temperatures, allowing to validate the reaction conditions and retain them for further investigations.

### Determination of the analytic limit of detection of the *Strumpellin* HRM PCR assay

The analytic limit of detection of the developed HRM PCR assay was determined by 8 times 10-fold dilution of DNA samples (20 ng to 2 fg) of representative strains corresponding to *L. major*, *L. infantum*, *L. tropica*. The mean Tm calculated using the Tm Calling software showed a slight variation of the Tm values (*L. infantum* Tm = 88.42 ± 0.03 °C, *L. major* Tm = 90.05 ± 0.03 °C, *L. tropica* Tm = 88.47 ± 0.15 °C). According to the normalized HRM curves (Fig 4B), the patterns were conserved regardless of the amount of DNA input in the PCR reaction (S5 Fig), even in the case of low Cp value (>30) (S6 Fig and S1 Table). The run was tested in triplicate. No amplification was observed with less than 2 pg of *Leishmania* DNA, regardless of the *Leishmania* species. This DNA amount could be converted to the putative number of *Leishmania* parasites according to the size of a sequenced *Leishmania* genome (i.e., 33.6 MB, 72.5 fg for *L. major* diploid genome plus an estimated 15% (10.9 fg) kDNA, making 83.4 fg of total DNA for a single parasite) [61]. According to these considerations, this HRM PCR assay was able to detect the equivalent of 24 *Leishmania* parasites.

### Validation of *Leishmania* species identification using the HRM PCR analysis

**Fig 5:**
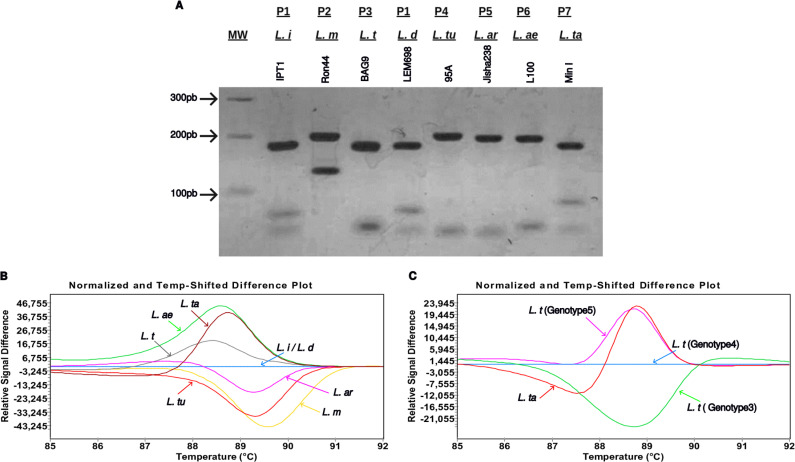
*Leishmania* species identification using ITS1-PCR RFLP and HRM PCR analysis. **(A)** Species/group of species - specific RFLP patterns (P) were obtained by digestion of ITS1-PCR products using the *HaeIII* restriction enzyme, and visualized on a 3% agarose gel electrophoresis. Seven patterns were observed corresponding to *L. infantum* (*L. i*) and *L. donovani* (*L.* d) (P1), *L. major* (*L. m*) (P2), *L. tropica* (*L. t*) (P3), *L. turanica* (*L. tu*) (P4), *L. arabica* (*L. ar*) (P5), *L. aethiopica* (*L. ae*) (P6) and *L. tarentolae (L. ta)* (P7). **(B)** Species/group of species-specific high resolution melting curves were obtained by the HRM PCR assay. The amplification reaction was performed on a Light Cycler 480 Instrument and analyzed by the Gene Scanning and Tm Calling software. Different melting curves representing different genotypes were observed, here corresponding to *L. infantum/L. donovani* (Genotype 1, blue curve, IPT1: Tm = 88.67 °C), *L. major* (Genotype 2, yellow curve, Ron44: Tm = 90.03 °C), *L. tropica* (Genotype 4, grey curve, BAG9: Tm = 88.44 °C), *L. aethiopica* (Genotype 6, green curve, L100: Tm = 88.05 °C), *L. arabica* (Genotype 7, pink curve, Jisha238: Tm = 89.75 °C), *L. turanica* (Genotype 8, red curve, 95A: Tm = 89.67 °C), *L. tarentolae (L. ta)* (Genotype 10, brown curve, Min I: Tm = 88.17 °C). **(C)** Identification of different *L. tropica* genotypes using the HRM PCR assay. The amplification of representative strains of *L. tropica* using the HRM PCR showed 3 different melting curves: one specific melting curve for *L. tropica* Genotype 3 (green curve, Tm = 88.98 ± 0.15 °C: representative strain AM: Tm = 89.19 °C, CJ: Tm = 89.01 °C, L0920: Tm = 88.9 °C, Gabai159: Tm = 88.83 °C), Genotype 4 (blue curve, Tm = 88.40 ± 0.03 °C, representative strain Bumm30: Tm = 88.43 °C, BAG9: Tm = 88.44 °C, Bumm30: Tm = 88.43 °C, A sinai III: Tm = 88.36 °C, Rachnan: Tm = 88.38 °C), Genotype 5 (pink curve, LA28: Tm = 88.14 °C), Genotype 10 (red curve, Min I: Tm = 88.17 °C).

To further validate the *Strumpellin* HRM PCR assay and establish the melting temperatures and curves of *Leishmania* species, our developed assay was applied to 73 *Leishmania* DNAs that have a range of geographical, host or clinical origins. They correspond to well documented (N = 46) or newly isolated (N = 27) parasites assigned to *L. major* (N = 34), *L. infantum* (N = 18), *L. tropica* (N = 13), *L. donovani* (N = 4), *L. turanica* (N = 1), *L. arabica* (N = 1), *L. aethiopica* (N = 1), *L. tarentolae* (N = 1), by ITS1-PCR RFLP or other identification methods. Seven ITS1-PCR RFLP patterns corresponding to *L. infantum (L. i)* and *L. donovani* (*L. d*) (P1), *L. major (L. m)* (P2), *L. tropica (L. t)* (P3), *L. turanica (L. tu)* (P4), *L. arabica (L. ar)* (P5), *L. aethiopica (L. ae)* (P6), *L. tarentolae (L. ta)* (P7) were observed (Fig 5A).

To validate the digestion patterns obtained via ITS1-PCR RFLP, representative ITS1-PCR products corresponding to distinct RFLP profiles were sequenced and analyzed. The BLAST analysis confirmed the species assignment and *in silico* digestion patterns of these sequences were generated. The predicted digestion profiles closely matched the experimental ones, with the exception of the smallest bands (20–24bp), which were not visible on the 3% agarose gel. Overall, the predicted RFLP patterns corroborated the experimental findings, further confirming the accuracy of the results (S7 Fig).

Nine different melting curves profiles were generated by HRM PCR for the DNAs of this parasite panel as expected from the sequence analyses (Fig 5B). One was observed for each of the *L. major*, *L. turanica, L. arabica, L. aethiopica* and *L. tarentolae* species. All the *L. major* strains (N = 34) shared the same curve profile. A fifth curve was observed for the different strains belonging to the different species of the *L. donovani* complex (N = 22) (*L. infantum, L. donovani*) including the ones synonymized to them (*L. archibaldi* (*L. d*)*, L. chagasi* (*L. i*)). Three different curve profiles were revealed for *L. tropica* DNAs (Fig 5C). The first curve (green) corresponds to 4 *L. tropica* strains one from Middle East (Gabai159) and 3 isolates from North Africa (AM, CJ, L0920), the second (blue) was assigned to 8 *L. tropica* strains from different Middle Eastern and Asian countries (BAG9, L75, Rachnan, K001, Bumm30, BAG17, SAF-K27, A Sinai III) and the third (pink) was observed with a strain originating from Southern Europe (LA28). These patterns correspond respectively to 3 genotypes (G3, G4, G5).

Furthermore, the melting temperature measured by Tm Calling software were specific to each genotype, explaining the differences in the melting curves. The melting temperatures were as follows: 88.58 ± 0.07 °C for *L. infantum* (IPT1, Dr13) and *L. donovani* strains G1 (L1005, LEM698), 90.04 ± 0.03 °C for *L. major* strains G2 (Ron99, Ron44, E12, E29), 88.05 °C for *L. aethiopica* G6 (L100), 89.75 °C for *L. arabica* G7 (Jisha238), 89.67 °C for *L. turanica* G8 (95A) and 88.17 °C for *L. tarentolae* G10 (Min I). For *L. tropica* three melting temperature were observed according to the genotypes: 88.98 ± 0.15 °C for *L. tropica* G3 (Gabai159, L0920, CJ, AM), 88.40 °C ± 0.03 °C for *L. tropica* G4 (BAG9, Bumm30, A Sinai III, Rachnan) and 88.14 °C for *L. tropica* G5 (LA28).

Despite the very similar Tm values of *L. tarentolae* and *L. tropica* (G5), at 88.17 °C and 88.14 °C respectively, the gene scanning program effectively distinguished between the two species. Unlike Tm calling, gene scanning can detect subtle variations in the DNA melting profiles, providing a more precise differentiation even between species with nearly identical Tm values (Fig 5C). Moreover, to confirm the specificity of the HRM PCR primers, one human blood DNA extracted from healthy donor was tested using KF4/KR4. No amplification was observed, indicating the absence of cross-reactivity between these primers and human DNA.

### Proof of principle validation of the HRM PCR analysis for the detection and identification of *Leishmania* species in clinical samples

To assess the relevance of the developed HRM PCR analysis assay for simultaneous detection and identification of *Leishmania* parasites in clinical samples, a total of 38 cutaneous samples collected during the 2010–2013 period from suspected CL patients were tested by the new assay and the results compared to those of direct smear examination routine diagnosis and ITS1-PCR RFLP analysis (Table 5). The collected samples correspond to patients aged from 2 to 81 years with 65.8% being in the 18–65 years range; the sex ratio of the patients was 0.52 with 34.2% males and 65.8% females. The patients were from different governorates in Tunisia, most of them being from Central Tunisia: Sousse (23.7%), Mahdia (18.4%), Sidi Bouzid (15.8%), Kairouan (13.2%) and Kasserine (13.2%), the others from other endemic governorates. They presented in their majority 1 cutaneous lesion (57.9%) against 42.1% with more than one: 2 (15.8%), 3 (10.5%), 4 (2.6%), 5 (5.3%), 6 (5.3%), and 7 lesions (2.6%) (Table 2 and S8 Fig).

**Table 5:** Detection and identification of clinical samples using ITS1-PCR RFLP and HRM PCR.

Patient	Year	DE	ITS1-PCR RFLP		HRM PCR		
			Detection	Identification	Detection	Identification	Cp
Pa1	2011	+	+	*L. m*	+	*L. m*	33.83
Pa2	2011	+	+	*L. m*	+	*L. m*	33.70
Pa3	2011	−	−	*−*	−	*−*	*−*
Pa4	2011	−	−	*−*	−	*−*	*−*
Pa5^+*^	2011	+	+	*L. m*	+	*L. m*	35.16
Pa6	2011	+	+	*L. m*	+	*L. m*	38.86
Pa7	2011	+	+	*L. m*	+	*L. m*	36.11
Pa8	2011	−	−	*−*	−	*−*	*−*
Pa9^+^	2012	+	+	*L. i*	+	*L. i*	29.90
Pa10	2013	+	+	*	+	*L. i*	33.51
Pa11	2011	−	−	*−*	−	*−*	*−*
Pa12	2011	−	−	*−*	−	*−*	*−*
Pa13	2011	−	−	*−*	−	*−*	*−*
Pa14	2011	+	+	*	−	*−*	*−*
Pa15	2011	−	−	*−*	−	*−*	*−*
Pa16	2011	+	+	*L. m*	+	*L. m*	35.57
Pa17	2011	+	+	*	+	*L. i*	31.45
Pa18	2011	+	+	*	+	*L. t*	41.16
Pa19	2011	−	−	*−*	−	*−*	*−*
Pa20^+*^	2010	+	+	*L. i*	+	*L. i*	27.34
Pa21	2010	+	+	*L. i*	+	*L. i*	32.82
Pa22	2010	+	−	*−*	+	*L. t*	42.71
Pa23	2011	−	−	*−*	−	*−*	*−*
Pa24^+^	2011	+	+	*L. m*	+	*L. m*	35.91
Pa25	2011	+	−	*−*	+	*L. m*	35.39
Pa26	2012	−	−	*−*	−	*−*	*−*
Pa27	2010	+	+	*L. t*	+	*L. t*	30.33
Pa28	2010	+	+	*L. m*	+	*L. m*	38.77
Pa29	2010	+	+	*L. m*	+	*L. m*	29.92
Pa30	2010	−	+	*L. m*	+	*L. m*	36.43
Pa31	2011	−	−	*−*	+	*L. m*	42.45
Pa32	2011	−	−	*−*	−	*−*	*−*
Pa33	2010	+	−	*−*	+	*L. m*	40.13
Pa34	2010	+	+	*L. m*	−	*−*	*−*
Pa35	2010	+	+	*L. m*	+	*L. m*	32.41
Pa36	2010	+	+	*L. m*	+	*L. m*	34.83
Pa37	2011	+	−	*−*	−	*−*	*−*
Pa38	2010	+	+	*L. m*	+	*L. m*	35.79

DE: Direct Examination, −: Negative, **+**: Positive, *: non conclusive but identification was confirmed by supplementary PCR assays as *L. infantum* (P10, P14, P17) and *L. tropica* (P18), *L. m*: *L. major*, *L. i*: *L. infantum*, *L. t*: *L. tropica,* ^+^: entirely sequenced *Strumpellin* PCR product, ^+*^: partially sequenced *Strumpellin* PCR product, Cp values were calculated using the Fit points software.

CL diagnosis was established by direct examination (DE) under microscope of Giemsa-stained smears of the lesion aspirates, considered as the gold standard test. Out of the 38 patients, 25 had amastigotes in their smears (65.79%) which confirmed the CL diagnosis. The ITS1-PCR and the HRM PCR positivities were 57.89% (22/38) and 63.16% (24/38), respectively. Among the 25 parasitologically confirmed CL cases, 21/25 (84%) were positive by ITS1-PCR and 22/25 (88%) by *Strumpellin* HRM PCR. Interestingly, 21/24 HRM positives had Cp > 30 confirming the sensitive detection of the parasite DNA by this assay (Table 5). One case (Pa37) was reported as positive by Direct examination but negative by both molecular tests (ITS1-PCR and HRM PCR). The B-globin PCR, used as internal control, also tested negative, indicated the quality/quantity of the extracted DNA was poor/insufficient to perform the PCR (S9 Fig).

**Fig 6:**
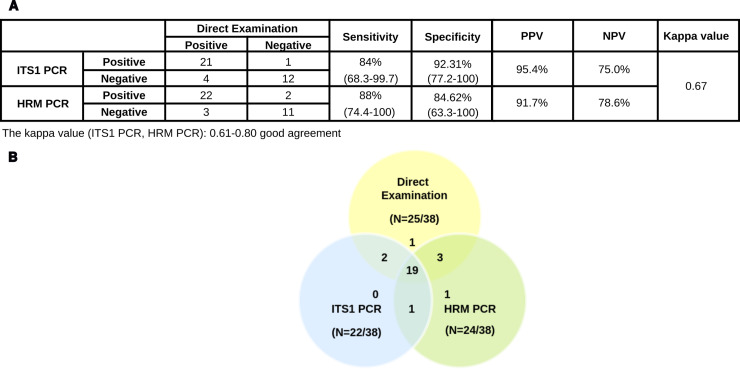
*Leishmania* parasites detection in suspected CL patients’ samples. DNAs of frozen cutaneous lesions aspirates sampled from 38 suspected patients were extracted and amplified by HRM- and ITS1-PCR assays. **(A)** Performances of the ITS1-PCR and HRM PCR using Direct Examination as gold standard for *Leishmania* parasites detection were measured. **(B)** Venn diagram representing *Leishmania* detection results obtained with ITS1-PCR, HRM PCR and Direct Examination.

The performances of the two molecular methods were evaluated and compared to the direct examination. The sensitivity and specificity of the HRM PCR assay for *Leishmania* infection detection were respectively 88% and 84.62% versus 84% and 92.31% for ITS1-PCR assay. The PPV and the NPV for the ITS1-PCR were 95.4% and 75%, and for HRM PCR 91.7% and 78.6% (Fig 6). The overall performance of HRM PCR in terms of sensitivity and NPV highlights its usefulness as a sensitive and reliable molecular diagnostic test. Good correlation and good agreement were observed between the different tests: HRM PCR and ITS1-PCR (κ = 0.67); Direct examination and ITS1-PCR (κ = 0.72); Direct examination and HRM PCR (κ = 0.71).

**Fig 7:**
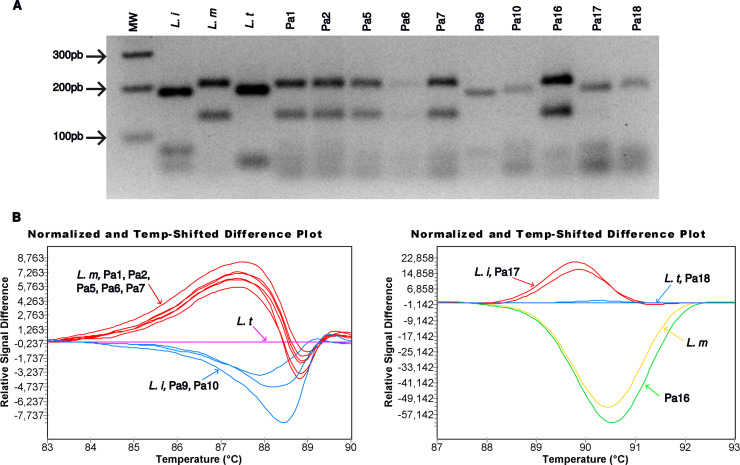
*Leishmania* parasites identification in suspected CL patients’ samples using ITS1-PCR RFLP and HRM PCR assays. DNAs of frozen cutaneous lesions aspirates sampled from 38 suspected patients were extracted by QIAamp DNA Mini Kit and amplified by HRM PCR assay and ITS1-PCR. **(A)** *Leishmania* parasites identification using ITS1-PCR RFLP: the amplicons of 3 reference *Leishmania* strains (*L. infantum* (*L. i*), *L. major* (*L. m*) and *L. tropica* (*L. t*)) and 10 samples (Pa1, Pa2, Pa5, Pa6, Pa7, Pa9, Pa10, Pa16, Pa17, Pa18) were digested with *HaeIII* restriction enzyme and visualized on 3% agarose gel electrophoresis, Mw: 100bp ladder. *Leishmania* parasites in samples Pa1, Pa2, Pa5, Pa6, Pa7 and Pa16 were identified as *L. major* and Pa9 as *L. infantum*, while in case of Pa10, Pa17 and Pa18 they could not be clearly assigned to *L. infantum* or *L. tropica*. **(B)** *Leishmania* parasites identification in patients’ samples using the HRM PCR assay. The amplified products of positive controls (*L. infantum* (*L. i*), *L. major* (*L. m*) and *L. tropica* (*L. t*)) and patients’ samples were analyzed using the Gene scanning and Tm calling softwares. The melting temperature and melting curves of the different samples were compared to those of the positive controls. The panel on the left illustrates the results of patient Pa1, Pa2, Pa5, Pa6, Pa7, Pa9 and Pa10; the causal agents were identified as *L. major (L. m* reference strain: LEM3171, Tm = 89.78 °C, CL patient Tm (Pa1) = 89.44 °C, Tm (Pa2) = 89.29 °C, Tm (Pa5) = 89.38 °C, Tm (Pa6) = 89.50 °C, Tm (Pa7) = 89.18 °C) as *L. infantum* (*L. i* reference strain: IPT1: Tm = 88.36 °C, CL patient Tm (Pa9) = 88.30 °C, Tm (Pa10) = 87.96 °C) while *L. tropica* reference strain: Gabai159: Tm = 88.69 °C. The panel on the right illustrates the results of Pa16, Pa17 and Pa18; the causal agents were identified as *L. major (L. m* reference strain: LEM3171: Tm = 91.07 °C, CL patient Tm (Pa16) = 90.81 °C), as *L. infantum* (*L. i* reference strain: IPT1: Tm = 89.60 °C, CL patient Tm (Pa17) = 89.41 °C), and *L. tropica (L. t* reference strain: Gabai159: Tm = 89.84 °C, CL patient Tm (Pa18) = 89.79 °C).

Parasites identification was performed by RFLP analysis of the positive ITS1-PCR products using *HaeIII* restriction enzyme. Species assignment was established according to the restriction profiles of *Leishmania* strains used as positive controls. In case of the HRM PCR assay, it was done by Gene scanning and Tm calling analyses, using the Light Cycler 480 software, determining the melting curves and temperature of the positive samples, then by comparing the melting curves and Tms to those measured for the reference *Leishmania* species DNAs, run with the samples’ DNAs. These results are illustrated in Fig 7.

The parasites were identified in a congruent manner by the two molecular methods (ITS1-PCR RFLP and HRM PCR). The positive lesion samples were found infected by *L. major* (N = 14 vs N = 16), *L. infantum* (N = 3 vs N = 5), *L. tropica* (N = 1 vs N = 3) using the ITS1-PCR RFLP and HRM PCR assays, respectively (Table 5). Importantly, the HRM PCR assay was able to identify all the detected parasites (24/24).

To confirm the species assignment of clinical samples via HRM PCR, DNA from 7 patients (Pa5, Pa7, Pa9, Pa10, Pa20, Pa24 and Pa25) was amplified and sequenced using the PCR MI5032F/R. However, only 4 of the 7 positive PCR products were successfully sequenced. Among them, Pa9 and Pa24 were entirely sequenced, covering the HRM PCR target region. These samples were identified as *L. infantum* and *L. major*, respectively, based on the Blast analysis conducted using TriTrypDB and sequence alignment that demonstrated species-specific SNPs (S10 Fig). Pa5 and Pa20 were only partially sequenced yielding 221bp and 252bp, respectively, from the 3’ end of the MI5032 fragment (S10 Fig). BLAST results for Pa5 showed an e-value of 2 E^−107^, a query cover of 100%, and 99% identity with LMJLV39_270023000 (*L. major*), while for Pa20, an e-value of 4 E^−123^, 99% query cover, and 99% identity with LINF_270023500 (*L. infantum*) were observed. This BLAST analysis confirmed the identification of Pa5 as *L. major* and Pa20 as *L. infantum*, as previously assigned through ITS1-PCR RFLP and HRM PCR (S10B Fig).

However only 18 out of the 22 positive samples were identified without ambiguity by the ITS1-PCR RFLP analysis. The 4 remaining ITS1 PCR products, corresponding to the Pa10, Pa14, Pa17, Pa18 samples, presented weak bands on agarose gel. Therefore, their restriction profiles were not conclusive and could not assign them to *L. tropica* or *L. infantum*. Pa10, Pa14, Pa17 were assigned to *L. infantum* and Pa18 to *L. tropica* by using other available PCR assays in the laboratory. Three of these CL samples were unambiguously assigned to *L. infantum* (Pa10, Pa17) and *L. tropica* (Pa18) using the HRM PCR assay, according to the melting curves obtained by the Gene scanning software and the Tm values by the Tm Calling software. Pa14 was not positive by this assay. Indeed, we can hypothesize that the low amount of parasite DNA present in the sample may be below the detection threshold, leading the inconsistent amplification results (ITS1-PCR, HRM PCR). In such cases, regardless of the copy number of the target, amplification may not occur as it depends on the probability to pick enough target DNA to amplify [61].

Among the negative cutaneous samples revealed by DE, two, Pa30 and Pa31, were found positive by HRM PCR assay as being infected by *L. major*. *Leishmania* species identification of Pa30 was confirmed by ITS1-PCR RFLP, Pa31 was negative by this assay.

## Discussion

Detection and identification of *Leishmania* species/group of species is essential for disease diagnosis, prognosis, patient’s management and treatment, epidemiology and control. Indeed, species identification could provide important data for decision making at the bed side or at programmatic and policy levels [62]. *Leishmania* lesions could atypically share features of several dermic affections such as sporotrichosis, mycobacterial infections, syphilis, carcinoma [63–65]. Moreover, susceptibility and tolerance to treatment depend on the infecting species [66].

Different technologies were used to determine species identity like PCR, PCR-RFLP, MLEE, MLST, DNA sequencing, where methods develop relevant sensitivity and specificity [56]. Technology advances now permit to aim for the development of simple, rapid, sensitive and specific DNA assays that could identify *Leishmania* species while detecting them. PCR High resolution melt curve analysis proved efficient in different diseases [13–15]. Studies have reported on the usefulness of this technology for identifying *Leishmania* at genus or species level in human samples using targets within genomic or mitochondrial DNA that are present in unique or high copy number in the genome [31,56,62].

Multicopy targets such as the kinetoplast DNA (kDNA) minicircles are considered as ideal targets for highly sensitive detection of *Leishmania* due to their presence on thousands copies per cell and for accurate discrimination between *Leishmania* species using conserved or variable regions [56]. Primers in the minicircles conserved region, distinguishing *L. major* from *L. donovani* and from *L. tropica* and *L. infantum* [67], were efficiently used in a prospective study using human skin samples on filter paper in Algeria [68], and on a collection of Giemsa stained smears of patients’ skin lesions in Iran [69]. Kinetoplast DNA was also targeted to discriminate by HRM between *L. (Leishmania)* and *L. (Viannia)* subgenera [29], and *Leishmania* species from New World in patients’ biopsies [30]. Other classical nuclear DNA targets were hit for HRM PCR based tests, such as the ITS1 region, the non-coding spacer DNA located between the SSU and LSU rRNAs, used to identify and quantify Old World *Leishmania* species (*L. infantum/L. donovani, L. major, L. tropica* and *L. aethiopica*) using samples from human patients, reservoir hosts and sand flies [26]. This method was also found most effective for chronic cutaneous leishmaniases (CCL) diagnosis on smears and allowed incrimination of *L. major* as another cause for CCL in Turkey [28]. The 7SL RNA, a component of the eukaryotic Signal Recognition Protein (SRP) ribonucleoprotein complex, is another repeated nuclear target for HRM PCR. Phylogenetic analyses of the sequences proved it adequate to differentiate *Leishmania* [70]; its usefulness to discriminate by HRM PCR between *L. major, L. tropica* and *L. infantum* parasites in human samples was reported [18]. The test also identified *Leishmania* parasites in human samples in Turkey [24], and demonstrated on Giemsa stained slides that in addition to *L. major*, *L. tropica* still occurs in Northeast Iran [17].

Protein coding sequences also constitute adequate targets for HRM PCR *Leishmania* detection and identification. Genes encoding for the *Heat* *shock* *protein* 70 (*Hsp*70) family were used to set an HRM PCR assay that discriminates between 7 species in Brazil and 3 in the Eurasian/African region, and another was designed for the species of *L.* (*Viannia*) subgenus [19]. One of these pairs was also used to identify *Leishmania* parasites in patients having cutaneous and mucocutaneous lesions, allowing the first time report in Paraguay of *L. amazonensis* and *L. guyanensis* leishmaniases [20]. *Hsp70* sequences were also successfully used to discriminate *Leishmania donovani* from *Leptomonas seymouri* that coexist in clinical samples from India and Sri Lanka, when kDNA minicircle HRM PCR and sequence analyses failed to do it [21].

In addition to their accuracy in discriminating *Leishmania* taxa, multicopy DNA targets offer an increased sensitivity of detection, but could be prone to sequence variations within taxa which could affect the consistency of the profiles for a given species as is the case for ITS1 [26] or *Hsp70* [19,71–73]. Unique copy genes (UCG) also showed promise in design of HRM PCR assays for the accurate *Leishmania* detection and identification. This is the case for instance of *AAP3*, gene encoding for the *amino acid permease 3*, which proved its ability to discriminate up to 11 *Leishmania* species (*L. donovani, L. infantum, L. major, L. tropica, L. mexicana, L. amazonensis, L. braziliensis, L. guyanensis, L. lainsoni, L. naiffi and L. shawi*) from Old and New World using 3 HRM PCR assays according to a proposed scheme [31]. Association of different DNA targets within an algorithm to support diagnosis, eco-epidemiology or genotyping was also observed in HRM based studies using multicopy or UCG sequences [19,71,72]. This has the advantage to overcome limits in the discriminatory potential of the targets by expanding the accuracy of the methods in identifying a range of species through a molecular typing approach.

Despite lower copy number that would affect as generally admitted sensitivity of detection, our hypothesis was that UCG present advantages to craft taxonomical reactivity and consistency, when these targets are under a selective pressure that maintains important parasite functions. This reasoning guided our choice of the *Strumpellin* encoding gene as an HRM target, a conserved UCG in different genomes of *Leishmania* species, *L. (Sauroleishmania)*, *Leptomonas*, *Crithidia* and *Endotrypanum* (TriTryp DB, Release 56, 15 Feb 2022), which previous phylogenetic studies revealed as a taxonomy marker particularly for Euglenozoa [33]. In addition, its loss in fungi and bacteria genomes makes it appropriate to enhance the specificity of the assay as these microorganisms could be found sur-infecting lesions and the absence of the target would minimize potential cross reactivity [74–76]. Thus, our aim was to prove its potential for detecting and identifying most *Leishmania* species infecting humans in the African and Eurasian regions. First, a bioinformatic study confirmed annotation of the protein with a somewhat different architecture from the human counterpart despite conservation of some motives, confirming the sequence divergence of the genes. The potential of the gene as a taxonomical marker in *Leishmania* was confirmed by multiple sequence alignments and phylogenetic analyses that indicated average 86.3% of pairwise similarity and 44.2% of identical sites, and a congruence of the phylogenetic tree topologies to *Leishmania* taxonomical classification. It was also found under negative selection pressure, which suggests the importance of the gene in *Leishmania* biology [77–79]. Then using a set of criteria for primers design, we defined a target region of the gene that bioinformatic and sequence analyses confirmed its adequacy as taxonomic marker that is under positive selection pressure, as described in different studies highlighting the usefulness of positive selection pressure to select targets for molecular diagnosis [80,81]. Thus, the KF4/KR4 sequence under positive selection pressure shared enough polymorphisms to differentiate and consistently identify the different *Leishmania* species encountered in the African and Eurasian regions using an HRM analysis approach.

Seventeen polymorphic sites were identified within the 120bp sequence amplified resulting in 10 sequence types identifying *L. donovani complex* (*L. donovani and L. infantum* species), *L. major*, *L. aethiopica*, *L. turanica*, *L. gerbilli*, *L. arabica*, *L. tarentolae* and *L. tropica* (3 types). Sequence analyses were confirmed by HRM PCR on a panel of *Leishmania* DNAs representing 7 of these species from various clinical or geographical origins, which resulted in different melting curves profiles and melting temperatures (Tm) which were consistently observed for the species where more than one DNA was tested. Interestingly, a single nucleotide difference observed between *L. arabica* and *L. turanica*, or *L. tropica* and *L. aethiopica*, or among the tested *L. tropica* strains reflected on both readouts of the HRM analysis, a well-known property that have made it an ideal method for studying SNPs [19,71,82]. Studies have so far consistently described ability of the developed assays to identify and differentiate among the 3 main species/taxa encountered in our part of the world: *L. major*, *L. tropica* and *L. infantum/ L. donovani* [18,28,69,83]. Few have focused on other species like the identification of *L. aethiopica* in addition to these 3 taxa [26], or *L. aethiopica* and *L. turanica* [60], or specifically addressed the differentiation of *L. donovani* from *Leptomonas seymouri* [21], encountered as a coinfecting parasite in human and vector samples [84]. Importantly, to the difference of another study, where similar melting curve profiles were observed within taxa despite the polymorphisms within their *Hsp70* target sequences [73], to each observed sequence types corresponded a *Leishmania* species, and experimental assessment showed they correspond to species-specific melting curves and Tm. Not surprisingly, we have identified 3 sequence types, melting curves and Tm for *L. tropica* species. Similar observations were previously made with HRM [18] or other methods and targets [85–87] explained by the high level of genetic diversity within this species in correlation with their geographic origin. The ability of our primers/target in this assay to differentiate multiple *Leishmania* species is quite remarkable, highlighting the strategic relevance of selecting the primers, in conserved gene regions that are flanking polymorphic sites across the different *Leishmania* taxa/species, in an UCG under negative selective pressures that ensured its adequacy as a reliable and consistent taxonomy marker. It will be important in future studies to assess if pathogenic (*L. aethiopica*) or non-pathogenic (*L. arabica*, *L. gerbilli*, *L. turanica, L. tarentolae*) species to humans (which reactivity and analysis were here assessed *in silico* or *in vitro* on one or 2 strains) could be consistently identified by their KF4/KR4 HRM analysis, thus confirming relevance of this assay to their identification. Likewise, the ability of KF4/KR4 primers to amplify (or not) other *Leishmania* species and subgenera (not tested here) should be assessed. Given the extent of sequence variation in the priming sites in case of *L. Viannia* species, for instance (Fig 3), one can speculate HRM PCR will not yield amplification products. Expanding the multiple sequence alignment of the targeted gene region to a range of species pertaining to kinetoplastid genera and subgenera in *Leishmania* highlighted a congruent diversity to taxonomy and underscored the relevance of *Strumpellin* (and its targeted region) as candidates of choice for inclusion into molecular typing or identification schemes of kinetoplastid organisms (Fig 3).

Analytical detection limits depend on the target copy numbers allowing detection of less than one parasite for targets like ITS1 rRNA gene [26] or *Hsp*70 [19], to 100–500 fg (1 to 5 parasites) in case of *AAP3* (present in one to 2 copies in *Leishmania*) [31]. In case of *Strumpellin*, we could detect up to 2 pg of *Leishmania* DNA input (24 parasites) and the species-specific melting curves were conserved, even in the case of low Cp value (>30). In addition, although the amplitude of the melting curve depended on the input amount in the reaction, there was not a notable variation in the measured Tm in these reactions (*L. infantum* Tm = 88.42 ± 0.03 °C*, L. major* Tm = 90.05 ± 0.03 °C*, L. tropica* Tm = 88.47 ± 0.15 °C). Tm variations resulting in overlaps due to DNA (parasite) load variations were described for other targets [19,31,73]. They are of comparable magnitude (±0.08 °C–0.16 °C) [31] to the one observed in this study (±0.03 °C–0.15 °C), when using similar amounts of *Leishmania* DNA input, under the same reaction condition. The HRM PCR is typically run under 96 well plate format which allows including DNAs of well characterized strains to the experiments as positive controls with varying input amounts to guide identification when the parasite load of the samples is unknown.

In addition, to overcome the Tm overlap limit, a parasite load interval with the best accuracy for the HRM typing scheme could be defined as previously described [73]. We cannot exclude here the occurrence of Tm overlap in the presence of a low parasite load. It will need to be addressed during the future clinical evaluation study of the KF4/KR4 HRM PCR on clinical samples. However, as shown on human samples, most of the positive samples had a Cp > 30 and they were unambiguously identified. In fact, this study brings proof of principle demonstration on the usefulness of this new method for accurately diagnosing the disease by defining its etiology.

Indeed, its ability to amplify and identify *Leishmania* DNA in lesion samples was demonstrated in this study with a sensitivity of 88% and a specificity of 84.62% in comparison with the direct examination of Giemsa-stained smears of the corresponding lesions. These performances were in good agreement with smear examination but also with those of another more classical molecular method, ITS1-PCR which had 84% sensitivity and 92.31% specificity. While HRM PCR’s specificity (84.62%) is somewhat lower than that of ITS1-PCR; its PPV, NPV values (91.7%, 78.6%) and sensitivity (88%) make it relevant when prioritizing detection of true positives while minimizing the risk of false negatives. Missed infections could be due either to the presence of inhibitors, the quality of the DNA (degraded), or the quantity of target DNA (low). The use of such test is especially important in disease surveillance and patient management, where early and accurate detection can prevent disease evolution and reduction of the unsightly scars. Importantly, parasites infecting all HRM positive samples were identified while parasite identity was ascertained only in 18 out of 22 (81.8%) ITS1-PCR+ samples. Four cases could not be identified without ambiguity by ITS1-PCR RFLP. There was, however, a perfect correlation in the species assignments with both methods for 18 of them, and the assignment made by HRM of the 4 cases was confirmed by another molecular method. Moreover, the assignment of four cases (Pa5, Pa9, Pa20, Pa24) was confirmed by sequencing the (MI5032F/R) PCR products, showing the observed species-specific SNPs to *L. infantum* and *L. major*. The value of an HRM PCR versus ITS1-PCR RFLP to confirm parasite identity in cutaneous human samples was also emphasized in other studies; a *7SL* HRM PCR was shown more sensitive than ITS1-PCR RFLP (59% vs 49%) and only 86% of the positive samples detected by ITS1-PCR RFLP were identified unlike the *7SL* HRM PCR where all samples were simultaneously detected and identified. The authors also reported the correlation (100%) of the results between the two methods [18]. Despite our target was an UCG, we obtained slightly different values of sensitivity and specificity compared to ITS1 assay, based on a multicopy sequence, with a good agreement between results of the 3 methods here compared. Indeed, in addition to target copy number, different factors can influence the PCR yield such as target length, target sequence content, primer characteristics (GC content, secondary structure, primers interaction with the background DNA [88–92]. It is also known that HRM technology, as it uses fluorescence for end point analysis, is more sensitive than agarose gel analysis that relies on eye observation under UV light of ethidium bromide- stained bands [93]. Moreover, multicopy genes/targets could lead to a counterproductive interaction of the primers with distantly spaced priming sites (e.g., the forward primer on repeat 1 and the reverse primer on repeat n), which may reduce the PCR yield.

Various HRM assays were validated for use in field samples to detect and identify infections in reservoirs or vectors in good agreement with ITS1-PCR RFLP that also demonstrated their superiority in identifying all the samples amplified [18,93]. We did not attempt to apply the KF4/KR4 HRM PCR on field samples, which would be done in future studies should its application in field studies be considered.

Microscopic detection of *Leishmani*a parasites is the conventional method commonly used in endemic regions to confirm diagnosis. However, its sensitivity depends essentially on the technical expertise of clinicians and the disease stage, the type of sampling, the location on the lesion, the person collecting the sample, and whether the sample is fresh and the slide properly prepared. Moreover, microscopic examinations do not allow assignment of the amastigotes to a given species, so far only achieved using DNA assays. In Tunisia like in many African and Middle Eastern countries, cutaneous leishmaniases represent a complex disease group with diverse eco-epidemiological situations with the implication of three *Leishmania* species or complexes at least (i.e., *L. major, L. infantum/L. donovani* and *L. tropica*). We have demonstrated here the ability of KF4/KR4 PCR to accurately detect and identify *Leishmania* species simultaneously from lesions aspirates collected from CL suspected patients. It thus appears as a method that could assist the diagnosis by microscopic examination in well-equipped health centers. Evaluation of its performances would need however to be made on a larger number of samples to ascertain this role. Its ability to identify a wide range of *Leishmania* species encountered in the African and Eurasian regions makes it a good candidate to include in a diagnosis algorithm for cutaneous leishmaniases, in endemic countries or for travel medicine purposes. Moreover, as an additional feature that makes KF4/KR4 HRM a promising method to support CL diagnosis, detection and identification were simultaneously done at the end of the amplification process (i.e., upon 1h30) while it needs at least two workdays to achieve identification of the *Leishmania* upon their detection by ITS1-PCR RFLP. Notable also are the features of HRM technology in this respect, the system is closed obviating needs for additional steps, also reducing manipulation of the amplification products and subsequent errors or carryover contaminations.

The development and implementation of a new diagnostic test in clinical practice could reply to one of three fundamental roles: used as screening test, replace an existing test or complement an existing test [94]. Moreover, molecular diagnostic algorithms are recommended for accurate diagnosis of infectious diseases; studies have demonstrated the utility of diagnostic algorithms for CL for accurate species identification [95,96] and for Visceral Leishmaniasis to reduce false or late diagnosis [97–99]. Our KF4/KR4 HRM PCR could replace or be used as a complementary test to enhance diagnosis accuracy precising etiology of cutaneous leishmaniases notably in well-equipped health centers. A more comprehensive diagnostic algorithm could be proposed, consisting on our KF4/KR4 HRM PCR specifically developed for Old World species as the first step, followed by already published HRM tests or newly developed assays based on *Strumpellin,* specific to New World species, as a second step, helping to avoid misdiagnosis in imported cases that would be suspected by the travel history of the patients.

## Conclusion

In conclusion, we demonstrated that *Strumpellin* is an adequate target for HRM method development to detect and identify *Leishmania* parasites. The KF4/KR4 HRM PCR could be considered as a good alternative for the diagnosis of cutaneous leishmaniases in the African and Eurasian regions. It works as a closed tube system avoiding crossover contamination and additional post- amplification handling (and potential operator errors) obviating the need for electrophoresis and subsequent analyses to identify the parasites. It is therefore faster and less costly than the other more classical molecular methods involving PCR amplification (PCR-RFLP, MLST, DNA sequencing), and seems more accurate than ITS1-PCR RFLP in identifying the causal agents. It is also well adapted to analyze multiple samples in parallel as it is processed in 96 or 384 wells plates. Its performances are in good agreement with microscopy or ITS1-PCR RFLP which make it a good candidate to assist CL diagnosis in well-equipped diagnosis centers.

## Supporting information

S1 Fig Schematic structure of aligned Human and *Leishmania Strumpellin* proteins. Proteins sequences were retrieved from NCBI (*KIAA0196*) and TriTrypDB (release 56, 15 Feb 2022; *LmjF27.1660*) corresponding respectively to human and *Leishmania Strumpellin* and aligned using Geneious Software (Geneious v.3.6.2). The schematic structure of the human *Strumpellin* protein (N-terminal, spectrin repeat and C-terminal domains) positions were identified as described by Clemen et al., 2010 [33]. The schematic structure of the *Leishmania Strumpellin* protein was identified according to InterPro program. It confirmed the *Strumpellin* family and predicted 5 domains (cytoplasmic domain (1175–1292), coil (534–554), disorder prediction (1118–1147), transmembrane region (1157–1174) and non cytoplasmic domain (1–1156)). The grey box corresponds to the location of the selected target (320–359) used for the development of the HRM PCR.
(TIF)

S2 Fig Synteny map for the *LmjF.*27.1660 gene in 18 species (15 *Leishmania* species (N = 25), 1 *Leptomonas* species (N = 1), 1 *Endotrypanum* species (N = 1) and 1 *Crithidia* species (N = 1)) available in TriTrypDB database. Each species was represented by one genomic sequence. Each gene is represented by an arrow showing the direction of transcription. The *LmjF.27.1660* highlighted (yellow) is conserved in all analyzed genomes.
(TIF)

S3 Fig Multiple sequence alignment of the *Strumpellin* coding sequences performed by Geneious v.3.6.2 program. Twenty-nine sequences were extracted from TriTrypDB database corresponding to *L. major* (LmjF.27.1660, LmjLV39_27, LmjSD75_27), *L. gerbilli* (LgeLEM452_27), *L. arabica* (LarLEM1108_27), *L. aethiopica* (LaeM147_27), *L. tropica* (LtrL590_27), *L. donovani* (Ld27_V01s1, CM002167.1, LR812647, LdLV9_27_V1_Pilon, CP029526), *L. turanica* (LtuLEM423_27), *L. infantum* (LinJ.27), *L. mexicana* (LmxM.27), *L. amazonensis* (KE391750.1), *L. tarentolae* (BLBS01000037, LtaP27), *L. braziliensis* (LbrM2903_27, LbrM.27, LbrM.27_v4_pilon), *L. panamensis* (LpaM13_27, CP009396), *L. enriettii* (LenLEM3045_27), *L. martiniquensis* (LMARLEM2494_27), *Endotrypanum* (EmoLV88_27), *Crithidia* (CfaC1_23), *Leptomonas* (Lsey_0015, LpyrH10_07). Numbers along the top of the alignment refer to the position of the region in the entire gene alignment. Black boxes indicate differences according to the consensus.
(TIF)

S4 Fig Sequence analyses of the HRM PCR target using sequencing data of the MI5032F/R PCR products with focus on the KF4/KR4 fragment. PCR amplification products using MI5032F/R flanking the HRM PCR target were sequenced to compare and confirm the nucleotide sequence composition between and within *Leishmania* species. The sequence analyses of the 30 *Leishmania* strains (**) were performed using Geneious software. The different sequences were aligned with the reference sequences retrieved from TriTrypDB (*) corresponding to *L. infantum* (JPMC5), *L. donovani* (LV9), *L. major* (SD75.1)*, L. tropica* (L590), *L. aethiopica* (L147), *L. turanica* (LEM423) *L. arabica* (LEM1108), *L. gerbilli* (LEM452), *L. tarentolae* (BLBS01000037). The HRM PCR primers pair KF4/KR4 are represented as yellow arrows.
(TIF)

S5 Fig The analytical limit of detection of the *Strumpellin* HRM PCR assay was calculated by serial dilutions of the DNA (20 ng to 2 fg) of *L. infantum* (IPT1), *L. tropica* (BAG9) and *L. major* (LEM3171). **(A)** Amplification curves of serial dilutions of *L. infantum, L. tropica* and *L. major* DNAs. The graphs representing Cp values as function of the DNA input amount (in log) are reported for each species. They show a good correlation coefficient R^2^ (0.997), R^2^ (0.9683), R^2^ (0.993) for *L. infantum, L. tropica* and *L. major*, respectively. Cp values were calculated using the Fit points software, for *L. infantum*: Cp (20 ng) = 21.97, Cp (2 ng) = 25.94, Cp (0.2 ng) = 31.54, Cp (0.02 ng) = 36.39, Cp (0.002 ng) = 40.40; for *L. tropica*: Cp (20 ng) = 23.53, Cp (2 ng) = 28.86, Cp (0.2 ng) = 34.91, Cp (0.02 ng) = 40.49, Cp (0.002 ng) = 41.84; for *L. major*: Cp (20 ng) = 24.13, Cp (2 ng) = 27.93, Cp (0.2 ng) = 34.28, Cp (0.02 ng) = 37.89, Cp (0.002 ng) = 41.94. **(B)** Melt peaks in serial dilution of *L. infantum, L. tropica* and *L. major*. Tm values were calculated using the Tm calling software, for *L. infantum*: Tm (20 ng) = 88.41 °C, Tm (2 ng) = 88.44 °C, Tm (0.2 ng) = 88.41 °C, Tm (0.02 ng) = 88.38 °C, Tm (0.002 ng) = 88.49 °C; for *L. tropica*: Tm (20 ng) = 88.29 °C, Tm (2 ng) = 88.40 °C, Tm (0.2 ng) = 88.43 °C, Tm (0.02 ng) = 88.58 °C, Tm (0.002 ng) = 88.68 °C; for *L. major*: Tm (20 ng) = 90.08 °C, Tm (2 ng) = 90.01 °C, Tm (0.2 ng) = 90.04 °C, Tm (0.02 ng) = 90.06 °C, Tm (0.002 ng) = 90.10 °C. The HRM PCR was able to detect *Leishmania* DNA until 2 pg with the three *Leishmania* species.
(TIF)

S6 Fig Melting curves of serial dilutions of *L. infantum, L. tropica* and *L. major.
* **(A)** The analyses using the Gene scanning software showed the conservation of the melting curves of the 3 *Leishmania* species represented in red, pink and green for the *L. infantum* (IPT1), *L. tropica* (BAG9) and *L. major* (LEM3171), respectively. These curves can differentiate between these species. The mean Tm was calculated using the Tm calling software. **(B)** Melting curves of *L. infantum* (IPT1), *L. tropica* (BAG9) and *L. major* (LEM3171) at 0.2 ng of DNA. Cp (IPT1) = 31.54, Cp (BAG9) = 34.91, Cp (LEM3171) = 34.28. Ability of the melting curves to differentiate species was maintained despite the Cp values > 30.
(TIF)

S7 Fig Predicted digestion patterns of the ITS1-PCR RFLP using SnapGene (version 7.2.1) based on sequencing data. Reference strains: lane1: IPT1 (*L. i*), lane 2: Ron44 (*L. m*), lane 3: BAG9 (*L. t*), lane 4: LEM698 (*L. d*), lane 5: 95A (*L. tu*), lane 6: Jisha238 (*L. ar*), lane 7: L100 (*L. ae*), lane 8: Min I (*L. ta*), 3% agarose gel, MW: 100bp.
(TIF)

S8 Fig Clinical and epidemiological features of the collected cutaneous samples tested in this study (N = 38) during 2010–2013 period from the Parasitology department of the Farhat Hached University Hospital in Sousse (Tunisia). The figure illustrates the distribution of the patients according to their **(A)** Gender, F: Female, M: Male; **(B)** Age; **(C)** Number of skin lesions; **(D)** Geographical origin.
(TIF)

S9 Fig B-globin PCR assay of the clinical sample Pa37. B-globin PCR assay was performed and visualized on 1.5% agarose gel electrophoresis. Human DNA was used as positive control, Pa37: clinical sample, NC: negative control, Mw: 100bp ladder.
(TIF)

S10 Fig Sequence analyses of the HRM PCR target of clinical samples using sequencing data of the MI5032F/R PCR products with focus on the KF4/KR4 fragment. **(A)** PCR amplification products using MI5032F/R flanking the HRM PCR target were sequenced to compare and confirm the species assignment of the patients Pa5, Pa9, P20 and Pa24. Sequences were blasted using TriTrypDB and aligned using Geneious. Sequences corresponding to *L. major* (*L. m*, Friedlin), *L. infantum* (*L. i*, JPCM5), *L. tropica* (*L. t*, L590) were retrieved from TriTypDB and used as reference sequences. MI5032 and KF4/R4 targets are represented as blue and yellow boxes, respectively. **(B)** BLAST results, using TriTrypDB, for the four aligned sequences (Pa5, Pa9, Pa20, Pa24), including the Gene ID of the subject, E-value, query coverage (%), and identity (%).
(TIF)

S1 Table Tm values and Cp values corresponding to different amounts of *Leishmania* DNA in a representative assay. (PDF)
