# Supplementary figures and images for: A High Resolution Melting Analysis (HRM) PCR assay for the detection and identification of Old World Leishmania species
Source: PLoS Negl Trop Dis. 2024 Dec 23;18(12):e0012762. doi: 10.1371/journal.pntd.0012762 (PMC11684767; doi:10.1371/journal.pntd.0012762)

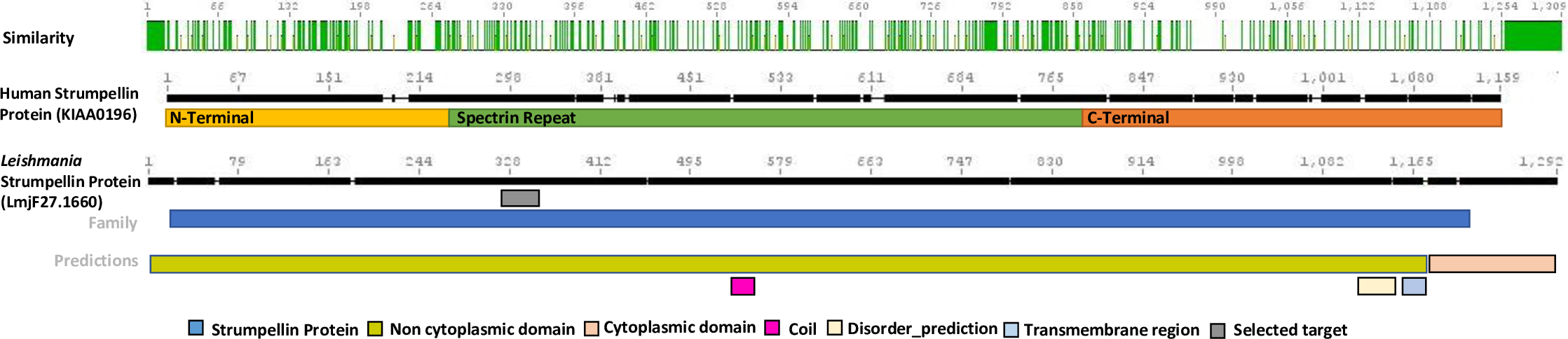

Supplement: S1 Fig — Proteins sequences were retrieved from NCBI (KIAA0196) and TriTrypDB (release 56, 15 Feb 2022; LmjF27.1660) corresponding respectively to human and Leishmania Strumpellin and aligned using Geneious Software (Geneious v.3.6.2). The schematic structure of the human Strumpellin protein (N-terminal, spectrin repeat and C-terminal domains) positions were identified as described by Clemen et al., 2010 [33]. The schematic structure of the Leishmania Strumpellin protein was identified according to InterPro program. It confirmed the Strumpellin family and predicted 5 domains (cytoplasmic domain (1175–1292), coil (534–554), disorder prediction (1118–1147), transmembrane region (1157–1174) and non cytoplasmic domain (1–1156)). The grey box corresponds to the location of the selected target (320–359) used for the development of the HRM PCR. (TIF) [file pntd.0012762.s001.tif]

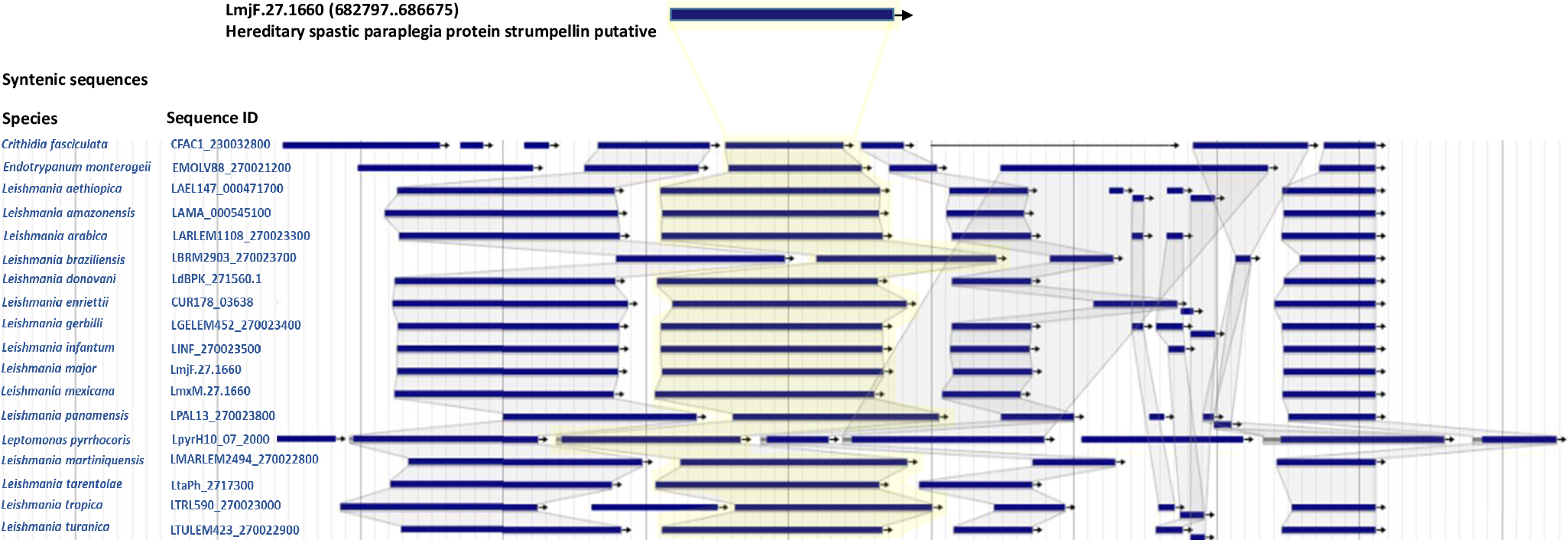

Supplement: S2 Fig — Each species was represented by one genomic sequence. Each gene is represented by an arrow showing the direction of transcription. The LmjF.27.1660 highlighted (yellow) is conserved in all analyzed genomes. (TIF) [file pntd.0012762.s002.tif]

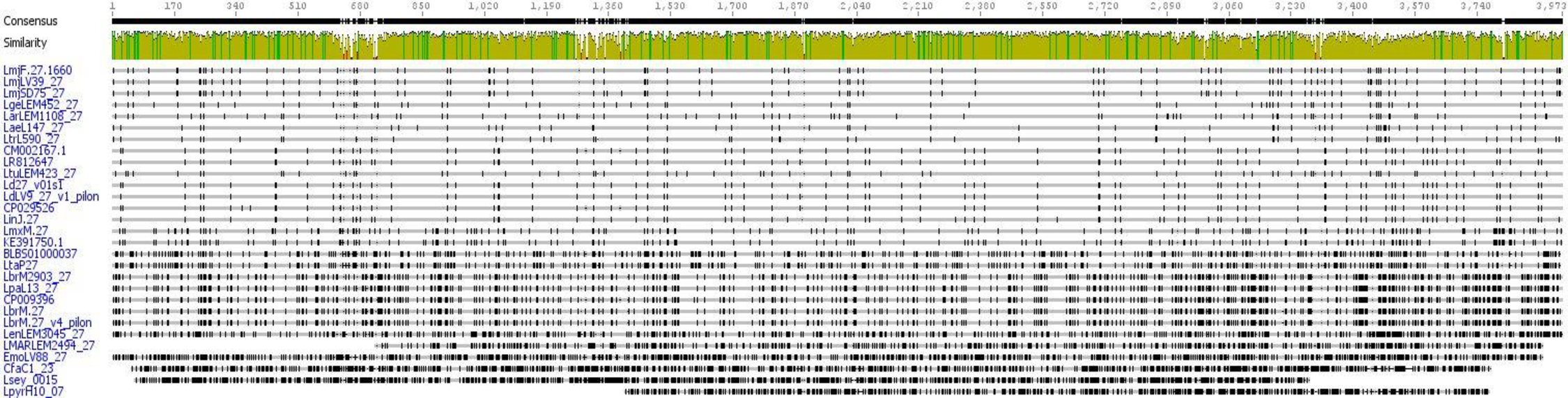

Supplement: S3 Fig — Twenty-nine sequences were extracted from TriTrypDB database corresponding to L. major (LmjF.27.1660, LmjLV39_27, LmjSD75_27), L. gerbilli (LgeLEM452_27), L. arabica (LarLEM1108_27), L. aethiopica (LaeM147_27), L. tropica (LtrL590_27), L. donovani (Ld27_V01s1, CM002167.1, LR812647, LdLV9_27_V1_Pilon, CP029526), L. turanica (LtuLEM423_27), L. infantum (LinJ.27), L. mexicana (LmxM.27), L. amazonensis (KE391750.1), L. tarentolae (BLBS01000037, LtaP27), L. braziliensis (LbrM2903_27, LbrM.27, LbrM.27_v4_pilon), L. panamensis (LpaM13_27, CP009396), L. enriettii (LenLEM3045_27), L. martiniquensis (LMARLEM2494_27), Endotrypanum (EmoLV88_27), Crithidia (CfaC1_23), Leptomonas (Lsey_0015, LpyrH10_07). Numbers along the top of the alignment refer to the position of the region in the entire gene alignment. Black boxes indicate differences according to the consensus. (TIF) [file pntd.0012762.s003.tif]

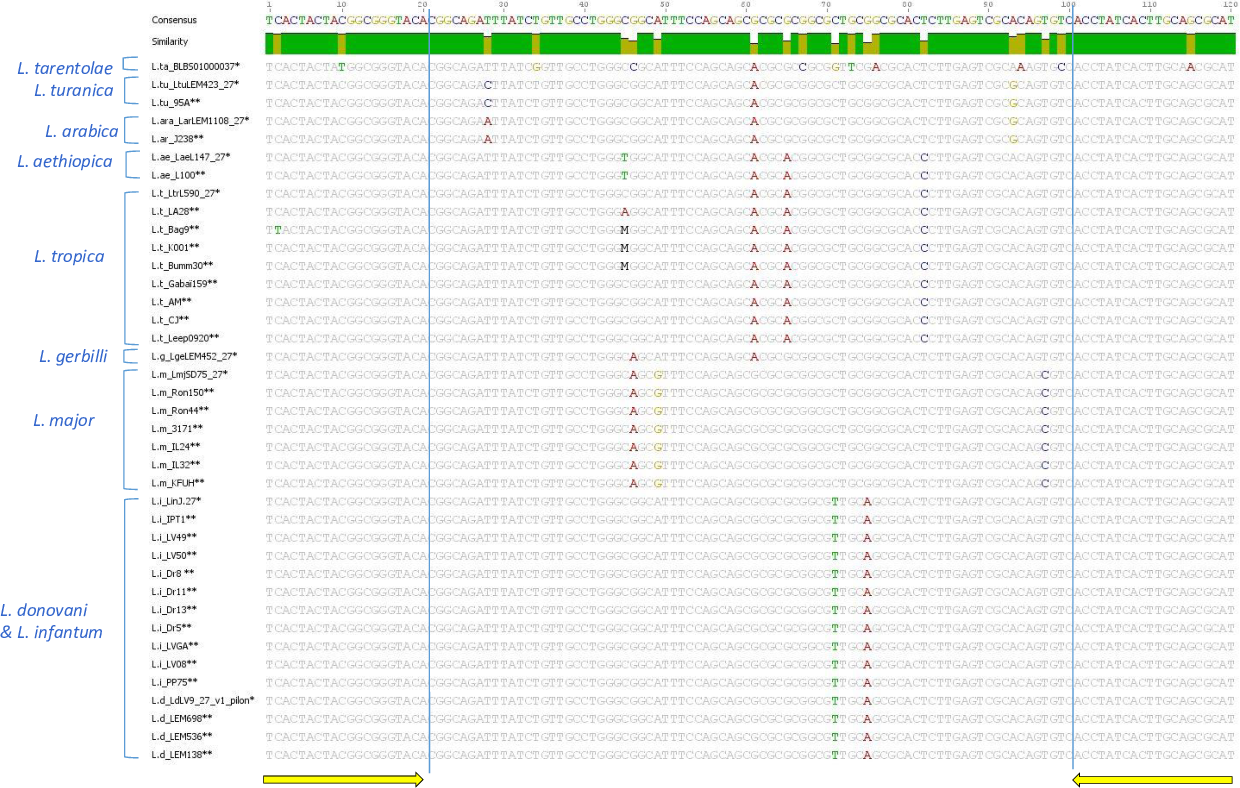

Supplement: S4 Fig — PCR amplification products using MI5032F/R flanking the HRM PCR target were sequenced to compare and confirm the nucleotide sequence composition between and within Leishmania species. The sequence analyses of the 30 Leishmania strains (**) were performed using Geneious software. The different sequences were aligned with the reference sequences retrieved from TriTrypDB (*) corresponding to L. infantum (JPMC5), L. donovani (LV9), L. major (SD75.1), L. tropica (L590), L. aethiopica (L147), L. turanica (LEM423) L. arabica (LEM1108), L. gerbilli (LEM452), L. tarentolae (BLBS01000037). The HRM PCR primers pair KF4/KR4 are represented as yellow arrows. (TIF) [file pntd.0012762.s004.tif]

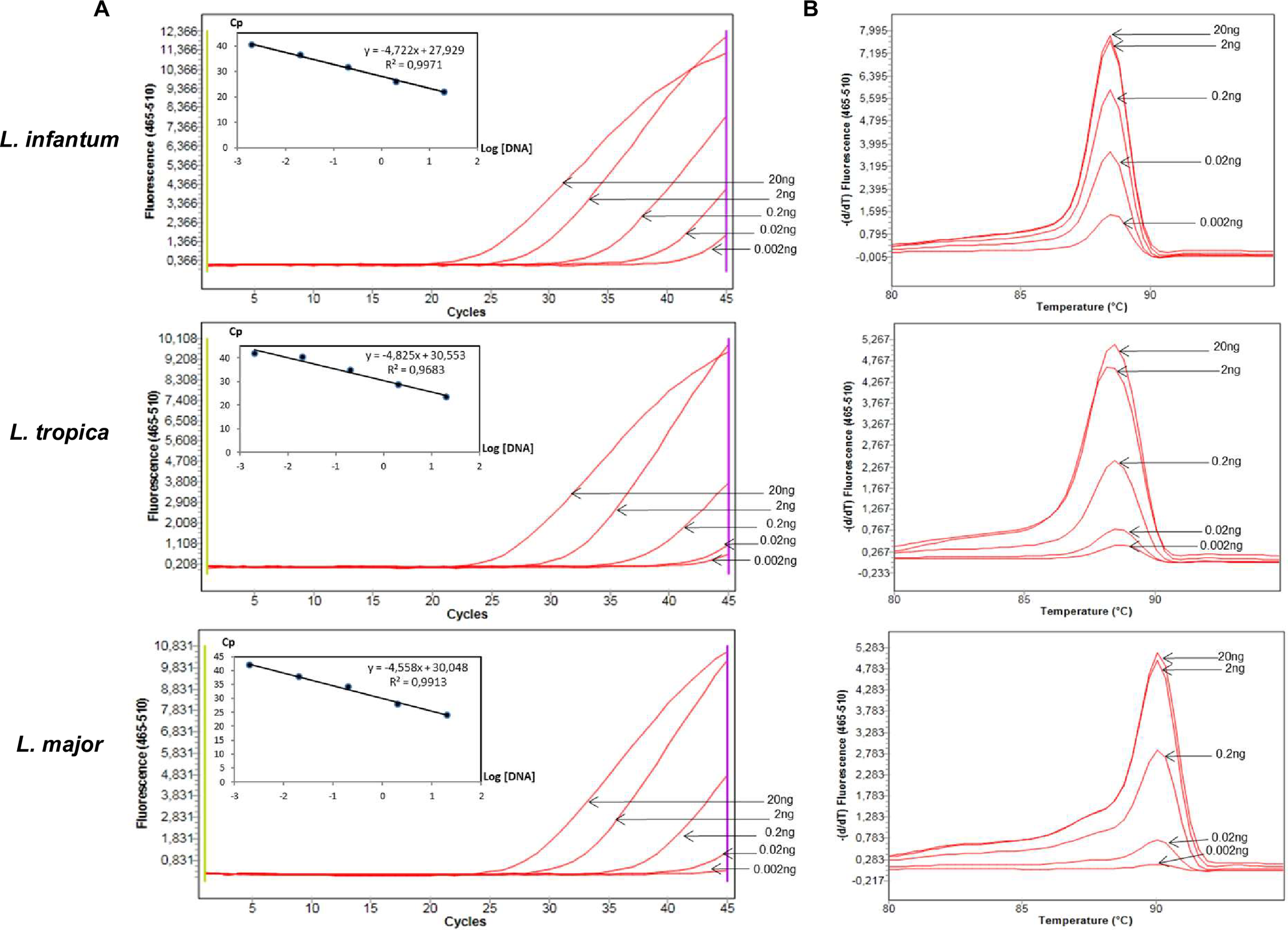

Supplement: S5 Fig — (A) Amplification curves of serial dilutions of L. infantum, L. tropica and L. major DNAs. The graphs representing Cp values as function of the DNA input amount (in log) are reported for each species. They show a good correlation coefficient R2 (0.997), R2 (0.9683), R2 (0.993) for L. infantum, L. tropica and L. major, respectively. Cp values were calculated using the Fit points software, for L. infantum: Cp (20 ng) = 21.97, Cp (2 ng) = 25.94, Cp (0.2 ng) = 31.54, Cp (0.02 ng) = 36.39, Cp (0.002 ng) = 40.40; for L. tropica: Cp (20 ng) = 23.53, Cp (2 ng) = 28.86, Cp (0.2 ng) = 34.91, Cp (0.02 ng) = 40.49, Cp (0.002 ng) = 41.84; for L. major: Cp (20 ng) = 24.13, Cp (2 ng) = 27.93, Cp (0.2 ng) = 34.28, Cp (0.02 ng) = 37.89, Cp (0.002 ng) = 41.94. (B) Melt peaks in serial dilution of L. infantum, L. tropica and L. major. Tm values were calculated using the Tm calling software, for L. infantum: Tm (20 ng) = 88.41 °C, Tm (2 ng) = 88.44 °C, Tm (0.2 ng) = 88.41 °C, Tm (0.02 ng) = 88.38 °C, Tm (0.002 ng) = 88.49 °C; for L. tropica: Tm (20 ng) = 88.29 °C, Tm (2 ng) = 88.40 °C, Tm (0.2 ng) = 88.43 °C, Tm (0.02 ng) = 88.58 °C, Tm (0.002 ng) = 88.68 °C; for L. major: Tm (20 ng) = 90.08 °C, Tm (2 ng) = 90.01 °C, Tm (0.2 ng) = 90.04 °C, Tm (0.02 ng) = 90.06 °C, Tm (0.002 ng) = 90.10 °C. The HRM PCR was able to detect Leishmania DNA until 2 pg with the three Leishmania species. (TIF) [file pntd.0012762.s005.tif]

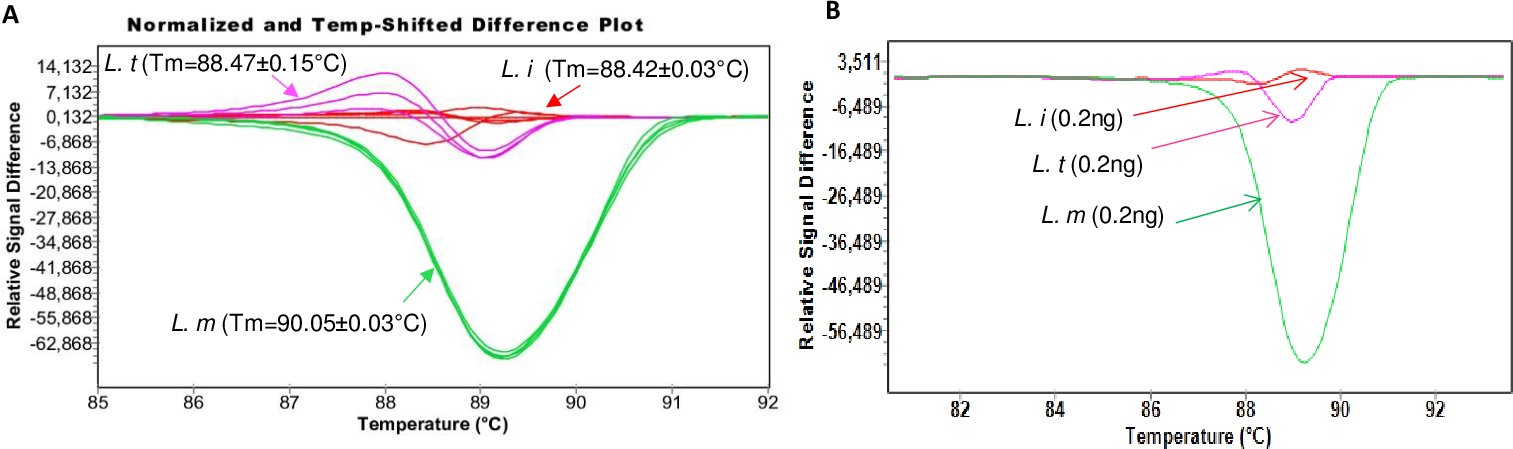

Supplement: S6 Fig — (A) The analyses using the Gene scanning software showed the conservation of the melting curves of the 3 Leishmania species represented in red, pink and green for the L. infantum (IPT1), L. tropica (BAG9) and L. major (LEM3171), respectively. These curves can differentiate between these species. The mean Tm was calculated using the Tm calling software. (B) Melting curves of L. infantum (IPT1), L. tropica (BAG9) and L. major (LEM3171) at 0.2 ng of DNA. Cp (IPT1) = 31.54, Cp (BAG9) = 34.91, Cp (LEM3171) = 34.28. Ability of the melting curves to differentiate species was maintained despite the Cp values > 30. (TIF) [file pntd.0012762.s006.tif]

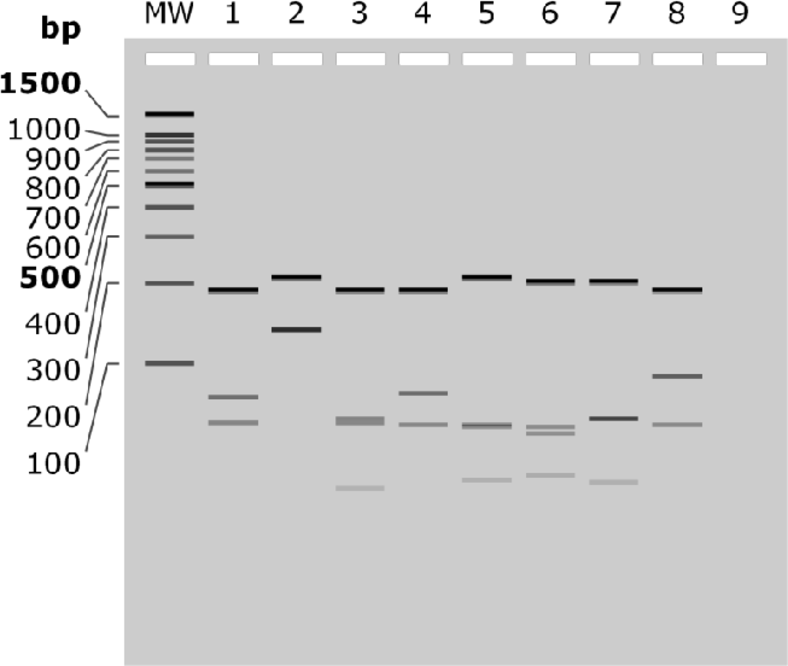

Supplement: S7 Fig — Reference strains: lane1: IPT1 (L. i), lane 2: Ron44 (L. m), lane 3: BAG9 (L. t), lane 4: LEM698 (L. d), lane 5: 95A (L. tu), lane 6: Jisha238 (L. ar), lane 7: L100 (L. ae), lane 8: Min I (L. ta), 3% agarose gel, MW: 100bp. (TIF) [file pntd.0012762.s007.tif]

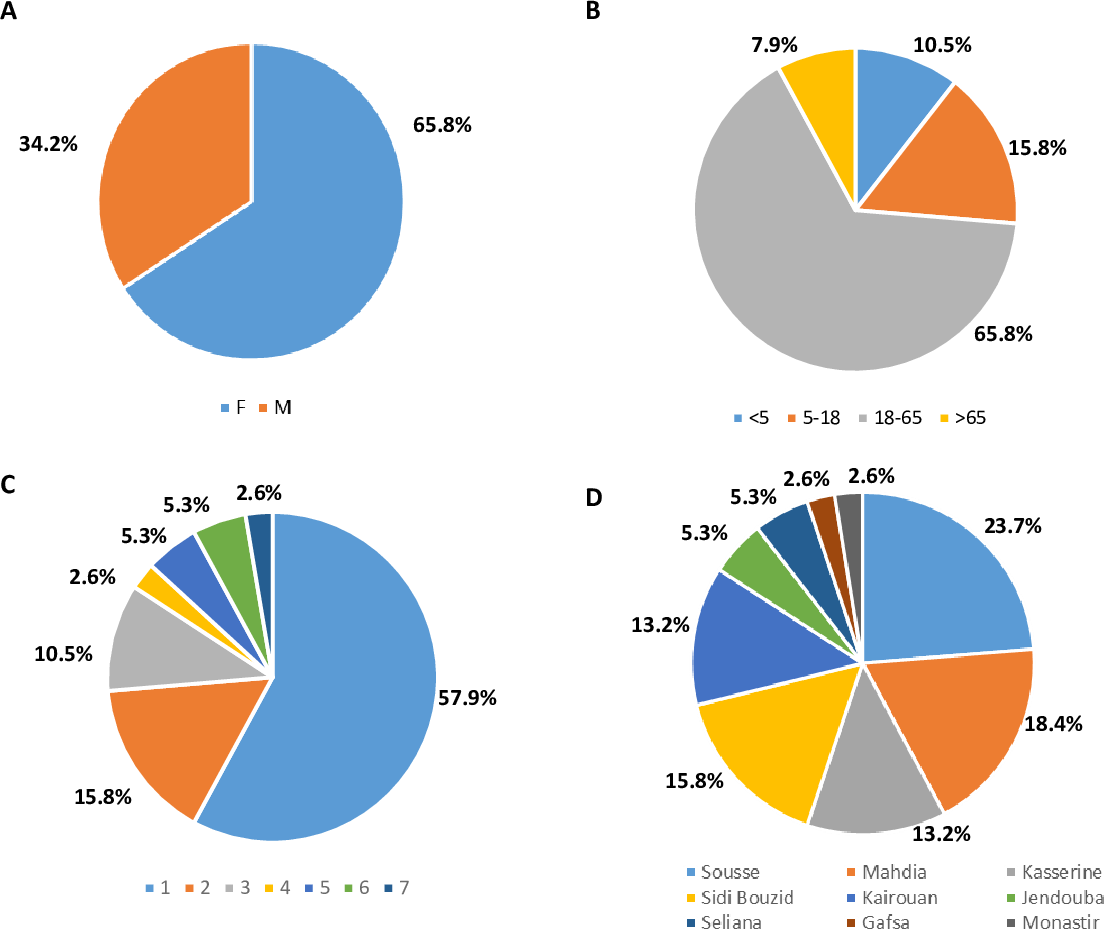

Supplement: S8 Fig — The figure illustrates the distribution of the patients according to their (A) Gender, F: Female, M: Male; (B) Age; (C) Number of skin lesions; (D) Geographical origin. (TIF) [file pntd.0012762.s008.tif]

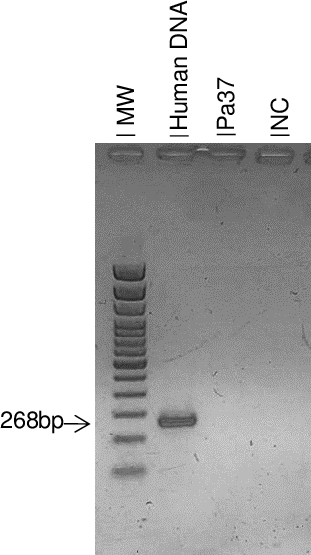

Supplement: S9 Fig — B-globin PCR assay was performed and visualized on 1.5% agarose gel electrophoresis. Human DNA was used as positive control, Pa37: clinical sample, NC: negative control, Mw: 100bp ladder. (TIF) [file pntd.0012762.s009.tif]

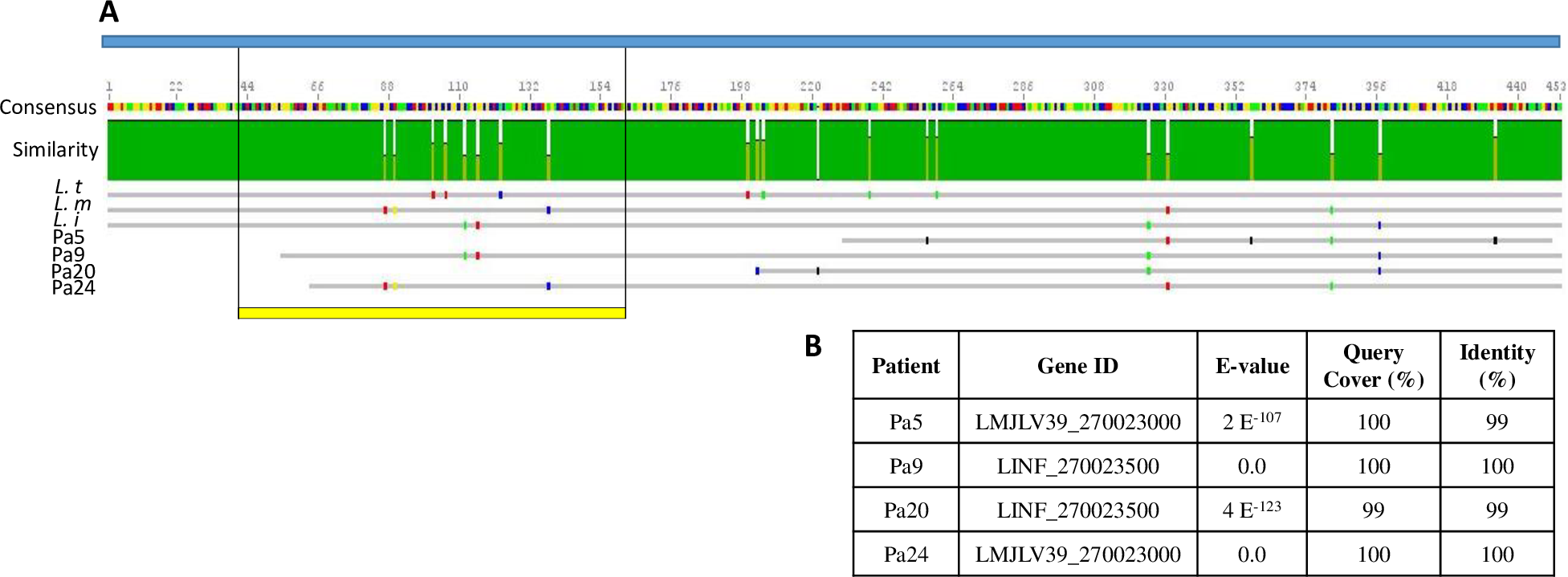

Supplement: S10 Fig — (A) PCR amplification products using MI5032F/R flanking the HRM PCR target were sequenced to compare and confirm the species assignment of the patients Pa5, Pa9, P20 and Pa24. Sequences were blasted using TriTrypDB and aligned using Geneious. Sequences corresponding to L. major (L. m, Friedlin), L. infantum (L. i, JPCM5), L. tropica (L. t, L590) were retrieved from TriTypDB and used as reference sequences. MI5032 and KF4/R4 targets are represented as blue and yellow boxes, respectively. (B) BLAST results, using TriTrypDB, for the four aligned sequences (Pa5, Pa9, Pa20, Pa24), including the Gene ID of the subject, E-value, query coverage (%), and identity (%). (TIF) [file pntd.0012762.s010.tif]
