# Supplementary material for: A High Resolution Melting Analysis (HRM) PCR assay for the detection and identification of Old World Leishmania species
Source: PLoS Negl Trop Dis. 2024 Dec 23;18(12):e0012762. doi: 10.1371/journal.pntd.0012762 (PMC11684767; doi:10.1371/journal.pntd.0012762)
Supplement: S1 Table — (PDF) [file pntd.0012762.s011.pdf]

S1 Table: Tm values and Cp values corresponding to different amounts of *Leishmania* DNA in a representative assay

DNA input	***L. infantum*** **(IPT1)**		***L. tropica*** **(BAG9)**		***L. major*** **(LEM3171)**	
	**Tm (°C)**	**Cp**	**Tm (°C)**	**Cp**	**Tm (°C)**	**Cp**
20ng	88.41	21.97	88.29	23.53	90.08	24.13
2ng	88.44	25.94	88.4	28.86	90.01	27.93
0.2ng	88.41	31.54	88.43	34.91	90.04	34.28
0.02ng	88.38	36.39	88.58	40.49	90.06	37.89
0.002ng	88.49	40.4	88.68	41.84	90.10	41.94
0.0002ng	88.42					

Tm(°C) and Cp were determined using Tm calling and Fit points programs, available in Light Cycler 480 software
